# Protocol for hybrid flux balance, statistical, and machine learning analysis of multi-omic data from the cyanobacterium *Synechococcus* sp. PCC 7002

**DOI:** 10.1016/j.xpro.2021.100837

**Published:** 2021-09-29

**Authors:** Supreeta Vijayakumar, Claudio Angione

**Affiliations:** 1 School of Computing, Engineering & Digital Technologies, Teesside University, Middlesbrough, North Yorkshire TS1 3BX, UK; 2 Centre for Digital Innovation, Teesside University, Middlesbrough TS1 3BX, UK; 3 Healthcare Innovation Centre, Teesside University, Middlesbrough TS1 3BX, UK

**Keywords:** Bioinformatics, Metabolism, Microbiology, Systems biology, Computer sciences

## Abstract

Combining a computational framework for flux balance analysis with machine learning improves the accuracy of predicting metabolic activity across conditions, while enabling mechanistic interpretation. This protocol presents a guide to condition-specific metabolic modeling that integrates regularized flux balance analysis with machine learning approaches to extract key features from transcriptomic and fluxomic data. We demonstrate the protocol as applied to *Synechococcus* sp. PCC 7002; we also outline how it can be adapted to any species or community with available multi-omic data.

For complete details on the use and execution of this protocol, please refer to Vijayakumar et al. (2020).

## Highlights

• Step-by-step instructions are provided for metabolic modeling and machine learning
• Any organism with a working GSMM and available transcriptomic data can be analyzed
• Analyzing multi-omic data provides a more complete picture of metabolism
• Machine learning algorithms reduce dimensionality and detect cross-omic features

## Graphical abstract

**Figure:**
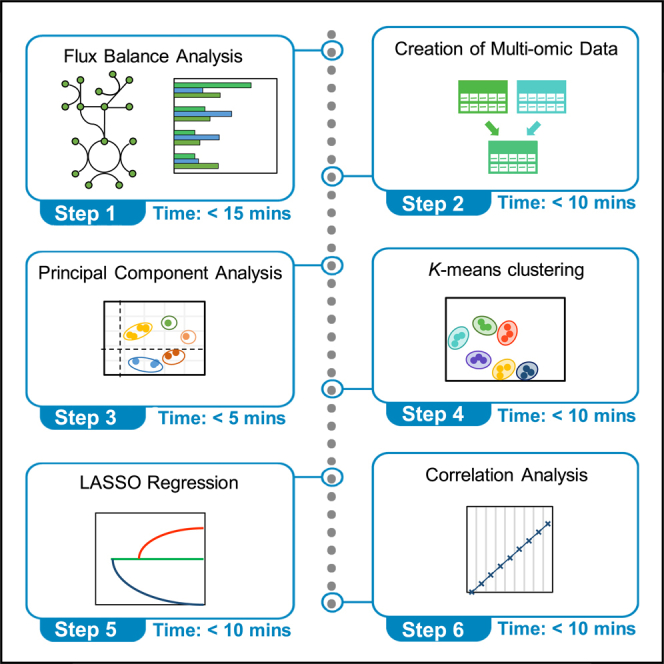

## Before you begin

The generation of a genome-scale view of metabolic activity is a useful step for many biological scientists, requiring the construction of a computational model that can be adapted to suit the purpose of each analysis by integrating omic additional data to simulate specific genetic or environmental conditions (Vijayakumar et al., 2018). Metabolic networks must be converted into a mathematical format that is both amenable to predictive modeling and able to effectively convey the functional state or behavior of the cell at a multi-systems level (Yurkovich and Palsson, 2015). To this end, genome-scale metabolic models (GSMMs) are mathematical representations of all known biochemical reactions and transmembrane transporters that occur within a living system. They provide a comprehensive view of all metabolic processes by recording and quantifying their flux, which can be defined as the rate of metabolic turnover or conversion of reactants into products (Palsson, 2015). Several methods for constraint-based reconstruction and analysis (COBRA) can be used to simulate flux through metabolic networks at the whole-genome scale (Bordbar et al., 2014). Among these, flux balance analysis (FBA) is a technique that utilizes linear programming to predict flux through all reactions in the metabolic network by locating a set of values in the solution space that best satisfies a given objective function representing the main metabolic goal for the cell (Reed, 2012; Dusad et al., 2020).

With the rapid advent of high-throughput technologies, supplementation of GSMMs with multi-dimensional omic data describing various levels of biological organization can provide the opportunity to trace molecular components across multiple functional states and record their interactions (Blazier and Papin, 2012; Ebrahim et al., 2016; Li et al., 2018). However, the quality of available experimental datasets can severely limit the predictive power of the model (Yurkovich and Palsson, 2018). To this end, there have been many recent studies that combine machine learning analyses with metabolic modeling (Nandi et al., 2017; Yaneske and Angione, 2018; Costello and Martin, 2018; Guebila and Thiele, 2019; Yang et al., 2019; Culley et al., 2020; Zhang et al., 2020a). Given the difficulty of extracting information from multi-omic datasets, machine learning algorithms serve to reduce dimensionality and elucidate cross-omic relationships (Cuperlovic-Culf, 2018). Additionally, machine learning algorithms and constraint-based models share complementary characteristics and common mathematical bases which make them compatible to be combined. On one hand, GSMMs can provide critical data in terms of stoichiometry and the genetic control of biochemical reactions. On the other hand, machine learning can deconstruct biological complexity by extracting relevant outputs from data. Together, they improve omic-based statistical and machine learning analyses by enriching the learning process with biological knowledge and refining phenotypic predictions (Zampieri et al., 2019; Volkova et al., 2020; Kim et al., 2020).

This protocol presents a series of steps that apply the principles of constraint-based metabolic modeling, multi-omic data integration and machine learning to analyze a genome-scale metabolic model of *Synechococcus* sp. PCC 7002 (summarized in Figures 2 and 3). Following this framework, the main stages comprise regularized flux balance analysis to observe flux response between growth conditions, as well as principal component analysis, *k*-means clustering, LASSO regression and correlation analysis to reduce dimensionality and extract key features from transcriptomic and fluxomic data. Through this synergistic approach, our goal is to achieve better characterization of metabolic activity across conditions by predicting the phenotypic response. We begin our protocol by presenting a brief summary of the software programs that must be installed prior to completing the main stages of analyses in Installation. Following this, we describe critical steps for the preparation of the chosen genome-scale metabolic model (GSMM) (Preparation of Metabolic Model) and the transcriptomic data (Preparation of Transcriptomic Data) for flux balance analysis. Preprocessing of transcriptomic data involves the conversion of reads per kilobase million (RPKM) into fold change values, which serves two purposes. First, each growth condition is normalized relative to the standard control within its dataset, allowing the integration of profiles relating to each growth condition during FBA. Second, calculating fold changes centered around 1 serves to facilitate comparisons between transcript and flux data when they are concatenated during later stages of analysis (PCA, LASSO and correlation).

Inputs and outputs for datasets used in each analysis are listed in Table 1.

**Table 1:** Data inputs and outputs

INPUTS	Outputs
**Preparation of metabolic model**	

**modelXML.xml**	**SynechococcusPCC7002.mat**
GSMM in XML format	GSMM in .mat format

**Preparation of transcriptomic data**	

**Dataset1.xlsx**	**transcripts.mat / transcriptsnew.csv**
Reads per kilobase million per mapped reads (RPKM) for gene transcripts in 16 experimental conditions and 3 standard controls	Transcript fold changes centered around 1, calculated by dividing RPKM values for experimental conditions by average RPKM of 3 standard controls
**Dataset2.xlsx**	
Reads per kilobase million per mapped reads (RPKM) for gene transcripts in 7 experimental conditions and 3 standard controls	

**Flux balance analysis**	

**transcripts.mat**	**all_atp_flux.mat / all_atp_flux.csv**
**SynechococcusPCC7002.mat**	Flux distribution recorded when conducting regularized bi-level FBA for the Biomass-ATP maintenance objective pair
**reaction_expression.mat**	**all_P1_flux.mat / all_P1_flux.csv**
Array defining connection between genes and reactions in the GSMM	Flux distribution recorded when conducting regularized bi-level FBA for the Biomass-Photosystem I objective pair
**pos_genes_in_react_expr.mat**	**all_P2_flux.mat / all_P2_flux.csv**
Array indexing the position of all genes within all reactions in the GSMM	Flux distribution recorded when conducting regularized bi-level FBA for the Biomass-Photosystem II objective pair
**ixs_genes_sorted_by_length.mat**	
Array indexing all genes by length (required when replacing genes with expression values)	
**Syn7002_IDs.mat**	
Gene IDs extracted from transcriptomic reads file	
**bounds.mat**	
Reaction names, indices and new values for lower and upper bounds to be adjusted in each growth condition prior to FBA	

**Creation of multi-omic dataset**	

**all_atp_flux.mat**	**ATPTF.mat / all_ATPTF.csv**
**all_P1_flux.mat**	Concatenated dataset of fold changes for gene transcripts and flux rates calculated with Biomass - ATP maintenance as objectives
**all_P2_flux.mat**	**P1TF.mat / all_P1TF.csv**
**transcripts.mat**	Concatenated dataset of fold changes for gene transcripts and flux rates calculated with Biomass - Photosystem I as objectives
	**P2TF.mat / all_P2TF.csv**
	Concatenated dataset of fold changes for gene transcripts and flux rates calculated with Biomass - Photosystem II as objectives

**Principal component analysis**	

**transcriptsnew.csv**	**contrib_transcripts.csv**
	Principal component contributions for gene transcripts
**all_ATP_flux.csv**	**contrib_all_atp_flux.csv**
	Principal component contributions for flux rates calculated with Biomass - ATP maintenance as objectives
**all_P1_flux.csv**	**contrib_all_p1_flux.csv**
	Principal component contributions for flux rates calculated with Biomass - Photosystem I as objectives
**all_p2_flux.csv**	**contrib_all_p2_flux.csv**
	Principal component contributions for flux rates calculated with Biomass - Photosystem II as objectives
**all_ATPTF.csv**	**contrib_all_ATPTF.csv**
	Principal component contributions for concatenated dataset of gene transcript and Biomass - ATP maintenance flux fold changes
**all_P1TF.csv**	**contrib_all_P1TF.csv**
	Principal component contributions for concatenated dataset of gene transcript and Biomass - Photosystem I flux fold changes
**all_P2TF.csv**	**contrib_all_P2TF.csv**
	Principal component contributions for concatenated dataset of gene transcript and Biomass - Photosystem II flux fold changes
	**ind_coord_all_atp_flux.csv**
	Principal component coordinates for Biomass - ATP maintenance flux data
	**ind_coord_all_p1_flux.csv**
	Principal component coordinates for Biomass - Photosystem I flux data
	**ind_coord_all_p2_flux.csv**
	Principal component coordinates for Biomass – Photosystem II flux data

**Pathway-level PCA**	

**contrib_all_atp_flux.csv**	**pathway_contrib_ATP.csv**
**contrib_all_p1_flux.csv**	Pathway-level PCA contributions to variance for Biomass - ATP maintenance fluxes
**contrib_all_p2_flux.csv**	**pathway_contrib_P1.csv**
**ind_coord_all_atp_flux.csv**	Pathway-level PCA contributions to variance for Biomass - Photosystem I fluxes
**ind_coord_all_p1_flux.csv**	**pathway_contrib_P2.csv**
**ind_coord_all_p2_flux.csv**	Pathway-level PCA contributions to variance for Biomass - Photosystem II fluxes

***K*-means clustering**	

**SynechococcusPCC7002.mat**	**silh_transcripts.fig / kmeans_transcripts.fig**
**Syn7002_IDs.mat**	Silhouette and *k*-means plots for transcript data
**transcripts.mat**	**silh_ATP.fig / kmeans_ATP.fig**
**all_atp_flux.mat**	Silhouette and *k*-means plots for Biomass - ATP maintenance fluxes
**all_P1_flux.mat**	**silh_P1.fig / kmeans_P1.fig**
**all_P2_flux.mat**	Silhouette and *k*-means plots for Biomass - Photosystem I fluxes
**ATPTF.mat**	**silh_P2.fig / kmeans_P2.fig**
**P1TF.mat**	Silhouette and *k*-means plots for Biomass - Photosystem II fluxes
**P2TF.mat**	**silh_ATPTF.fig / kmeans_ATPTF.fig**
	Silhouette and *k*-means plots for concatenated transcripts and Biomass - ATP maintenance fluxes
	**silh_P1TF.fig / kmeans_P1TF.fig**
	Silhouette and *k*-means plots for concatenated transcripts and Biomass - Photosystem I fluxes
	**silh_P2TF.fig / kmeans_P2TF.fig**
	Silhouette and *k*-means plots for concatenated transcripts and Biomass - Photosystem II fluxes

**LASSO regression**	

**transcripts_subset.mat**	**B_transcripts_nonzero.xlsx**
Subset of transcript data corresponding to available growth conditions	Non-zero fitted least-squares regression beta coefficients for LASSO conducted with gene transcripts (x) and growth rates (y).
**all_atp_flux_subset**	**B_ATP_nonzero.xlsx**
Subset of Biomass - ATP maintenance flux data corresponding to available growth conditions	Non-zero fitted least-squares regression beta coefficients for LASSO conducted with Biomass - ATP maintenance fluxes (x) and growth rates (y).
**all_p1_flux_subset**	**B_P1_nonzero.xlsx**
Subset of Biomass - Photosystem I flux data corresponding to available growth conditions	Non-zero fitted least-squares regression beta coefficients for LASSO conducted with Biomass - Photosystem I fluxes (x) and growth rates (y).
**all_p2_flux_subset**	**B_P2_nonzero.xlsx**
Subset of Biomass - Photosystem II flux data corresponding to available growth conditions	Non-zero fitted least-squares regression beta coefficients for LASSO conducted with Biomass – Photosystem II fluxes (x) and growth rates (y).
**ATPTF_subset**	**B_ATPTF_nonzero.xlsx**
Subset of concatenated transcript and Biomass - ATP maintenance flux data corresponding to available growth conditions	Non-zero fitted least-squares regression beta coefficients for LASSO conducted with concatenated gene transcripts and Biomass - ATP maintenance fluxes (x) and growth rates (y)
**P1TF_subset**	**B_P1TF_nonzero.xlsx**
Subset of concatenated transcript and Biomass - Photosystem I flux data corresponding to available growth conditions	Non-zero fitted least-squares regression beta coefficients for LASSO conducted with concatenated gene transcripts and Biomass - Photosystem I fluxes (x) and growth rates (y)
**P2TF_subset**	**B_P2TF_nonzero.xlsx**
Subset of concatenated transcript and Biomass - Photosystem II flux data corresponding to available growth conditions	Non-zero fitted least-squares regression beta coefficients for LASSO conducted with concatenated gene transcripts and Biomass - Photosystem II fluxes (x) and growth rates (y)
**Y2.mat**	
Growth rates corresponding to available growth conditions	

**Correlation analysis**	

**transcripts_subset.mat**	**corr_transcript_table.xlsx**
	Pearson correlation coefficients, P-values, lower and upper bounds according to the 95% CI calculated between gene transcripts (x) and growth rates (y)
**all_atp_flux_subset**	**corr_ATP_table.xlsx**
	Pearson correlation coefficients, P-values, lower and upper bounds according to the 95% CI calculated between Biomass - ATP maintenance fluxes (x) and growth rates (y)
**all_p1_flux_subset**	**corr_P1_table.xlsx**
	Pearson correlation coefficients, P-values, lower and upper bounds according to the 95% CI calculated between Biomass - Photosystem I fluxes (x) and growth rates (y)
**all_p2_flux_subset**	**corr_P2_table.xlsx**
	Pearson correlation coefficients, P-values, lower and upper bounds according to the 95% CI calculated between Biomass - Photosystem II fluxes (x) and growth rates (y)

**Pathway-level correlation analysis**	

**corr_ATP_table.mat**	**ATP_PCC_mean.mat**
	Mean absolute Pearson correlation coefficients calculated between Biomass - ATP maintenance fluxes (x) and growth rates (y) for each subsystem of the GSMM
**corr_P1_table.mat**	**P1_PCC_mean.mat**
	Mean absolute Pearson correlation coefficients calculated between Biomass - Photosystem I fluxes (x) and growth rates (y) for each subsystem of the GSMM
**corr_P2_table.mat**	**P2_PCC_mean.mat**
	Mean absolute Pearson correlation coefficients calculated between Biomass - Photosystem II fluxes (x) and growth rates (y) for each subsystem of the GSMM
	**all_corr_ATP.xlsx**
	PCC values calculated between Biomass - ATP maintenance fluxes (x) and growth rates (y) for all reactions within each subsystem
	**all_corr_P1.xls**
	PCC values calculated between Biomass - Photosystem I fluxes (x) and growth rates (y) for all reactions within each subsystem
	**all_corr_P2.xlsx**
	PCC values calculated between Biomass - Photosystem II fluxes (x) and growth rates (y) for all reactions within each subsystem

### Installation

**Timing: 1–2 h**

All installations can be run using Linux, Mac or Windows operating systems, but this protocol is mainly based on using the Windows platform. For full instructions on installing the COBRA Toolbox in Mac and Linux, we refer the reader directly to follow the steps provided at: https://opencobra.github.io/cobratoolbox/stable/installation.html.

1. If needed, install the latest version of MATLAB.
  a. The MATLAB programming language can be downloaded from https://uk.mathworks.com/downloads/web_downloads/. Following registration, a free 30-day trial can be requested from https://uk.mathworks.com/campaigns/products/trials.html.
  b. For a permanent installation, an associated license can be purchased for use by commercial or government organizations, degree-granting institutions, or individuals. Several universities and research organizations provide access to MATLAB through a centralized, campus-wide license.
2. Check if you have a working installation of git by typing \$ git --version in the Terminal (on Linux and macOS) or cmd (in Windows, not Git Bash). For the latest source release of git, check https://git-scm.com/downloads.
3. Download the latest version of the COBRA Toolbox and its compatible solvers from: https://opencobra.github.io/cobratoolbox/stable/installation.html. Alternative implementations of COBRA are listed in materials and equipment.
4. Install the Gurobi Optimizer from: https://www.gurobi.com/products/gurobi-optimizer/. This is required as a quadratic optimization solver during the regularized flux balance analysis steps. For a list of alternative solvers, see materials and equipment.

### Preparation of metabolic model

**Timing: 2 weeks to 1 month**

**Figure 1:**
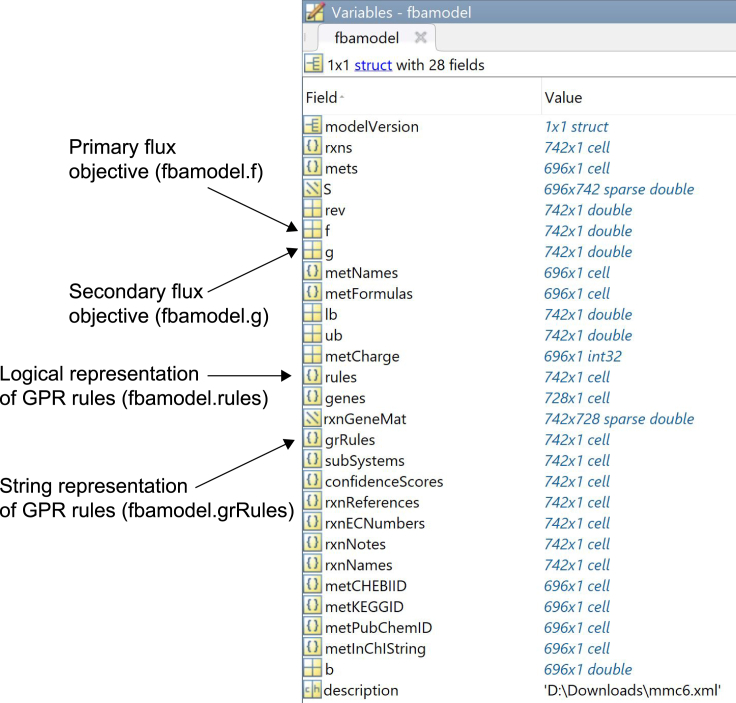
A list of all fields present in the *Synechococcus* sp. PCC 7002 GSMM (saved as *fbamodel.mat*).

Any organism with a baseline GSMM and available transcriptomic data can be analyzed using this protocol. The COBRA Toolbox is a popular module for constraint-based reconstruction and analysis of metabolic networks in MATLAB (Heirendt et al., 2019). In most cases, models are written in the Systems Biology Markup Language (SBML) to ensure compliance with the COBRA modules used for analysis (Keating et al., 2006). In this instance, we convert the model directly into .mat format for analysis in MATLAB using the COBRA Toolbox (the resulting model is shown in Figure 1).

Many GSMMs are publicly available in online repositories such as the Kyoto Encyclopedia of Genes and Genomes (KEGG) (Kanehisa et al., 2016), the Biochemical Genetic and Genomic (BiGG) knowledge-base (Norsigian et al., 2020), the BioCyc collection of pathway/genome databases (Karp et al., 2019), MetaNetX (Moretti et al., 2021) and the ModelSEED and PlantSEED databases (Devoid et al., 2013; Seaver et al., 2014). The preparation of these models for flux balance analysis involves the automated reconstruction of all metabolic reactions taking place in the organism, supplemented by the functional annotation of genes, metabolites and pathways. This is usually followed by extensive manual curation and gap-filling (Prigent et al., 2017), the extent of which is subject to the quality of the initial model reconstruction (Lieven et al., 2020). Furthermore, predictions obtained from GSMMs can be reconciled with *in vivo* findings and used to identify current gaps in our knowledge of metabolism (Mienda, 2017). However, there are often inconsistencies that must be reconciled between models and experimental data that would otherwise result in outcomes that are falsely predicted by the model (false positives) or experimentally observed outcomes that the model fails to predict (false negatives).

5. Create a genome-scale model for *Synechococcus* sp. PCC 7002 by converting model.xml into a .mat model in MATLAB:

% Add cobratoolbox and Gurobi directories to MATLAB path

addpath(genpath('C:\Users\xxxx\xxxx\cobratoolbox'))

addpath(genpath('C:\gurobi911'));

% Initialize the COBRA Toolbox

initCobraToolbox

% Create a .mat model from an XML model

fbamodel = readCbModel('modelXML.xml');

***Note:*** In order to relate genes, metabolites and reactions during FBA, the GSMM must contain a field of logical gene-protein-reaction (GPR) association rules. These rules record the involvement of every gene in every reaction of the metabolic network and must be adjusted when integrating new data that record differential gene expression under various conditions.

***Note:*** Although the field *fbamodel.rules* already exists within the model, running *compute_reaction_expression.m* creates the field *fbamodel.grRules* (a string representation of the GPR rules), which will be solved mathematically at the stage of omic data integration. As these new rules do not contain parentheses, it must be manually ensured that AND is solved before OR when substituting MIN and MAX respectively. This means that in the final expression, the MINs must be calculated before the MAXs. The function *associate_genes_reactions.m* called by *compute_reaction_expression.m* substitutes the ORs first (which become MAXs), and then the ANDs inside the MAXs. This generates an expression that first solves the ANDs (within an internal loop) and then solves the ORs (within an external loop).

6. Create new fields within the model for *grRules* and two flux objectives (*f* and *g*) that will be specified in flux balance analysis:

% Add new field for grRules

fbamodel = creategrRulesField(fbamodel)

% Remove field c that is used to specify a single flux objective

field = 'c';

fbamodel = rmfield(fbamodel,field)

% Create new fields f and g (whose length is equal to the number of reactions in the model)

to later specify pairwise primary and secondary flux objectives in the model

fbamodel.f = zeros(742,1);

fbamodel.g = zeros(742,1);

% Save the model in .mat format

writeCbModel(fbamodel,'format','mat','fileName','SynechococcusPCC7002.mat');

7. Match the parsing of gene IDs in the transcriptomic data with those listed in *fbamodel.genes*:

% Run the script compute_reaction_expression.m, which calls the function associate_genes_reactions.m in order to substitute the expressions AND and OR with MIN and MAX within fbamodel.grRules and creates the variables required for condition−specific flux balance analysis − i.e. pos_genes_in_react_expr, reaction_expression and ixs_genes_sorted_by_length

compute_reaction_expression;

**CRITICAL:** When parsing the strings within *grRules* (i.e., replacing AND and OR with MIN and MAX), it is essential to check the parentheses to ensure that the code runs correctly. Depending on the existing parsing rules for parentheses, it may be necessary to edit *associate_genes_reactions.m* to adjust the substitution loop according to the model in question.

**CRITICAL:** Steps 6 and 7 only apply when creating a new GSMM, as it must be ensured that a new *grRules* field is written in the model to link gene IDs in the omic dataset with those in the model. When applying the steps to a new model or data, it is important to ensure the consistency of gene names between external data and the GSMM, but modelers wishing to run the analysis for the *Synechococcus* GSMM only need to load the variables already saved in the code repository.

8. As stated previously, conducting manual curation of all model fields, including genes, reactions, metabolites and subsystems prior to performing FBA is necessary to ensure the verity of biological outputs. Particularly, subsystems within the model may be known by multiple names or annotated inconsistently. It is also possible, as in our case, that several reactions are assigned with multiple subsystems or even none at all. In the case of reactions, we create a new array of subsystem names that are modified to account for reactions classified by more than one subsystem:

% Create a cell array of subsystems

subsystems = fbamodel.subSystems;

% Merge the same names for amino acid metabolism into a single subsystem

old_aa = {'Amino Acid Metabolisms','Amino Acid Metabolism'};

new_aa = 'Amino acid metabolism';

subsystems = replace(subsystems,old_aa,new_aa);

% Merge the same names for exchange reactions into a single subsystem

subsystems = replace(subsystems,'Exchange Reaction','Exchange');

% Divide the names for lipid and cell wall metabolism into separate subsystems

subsystems = replace(subsystems,'Lipid and Cell Wall Metabolism', 'Lipid metabolism and Cell wall');

% Merge the same names for unassigned reactions into a single subsystem

old_none = {'None','Other'};

new_none = 'Unassigned';

subsystems = replace(subsystems,old_none,new_none);

subsystems(cellfun('isempty',subsystems)) = {'Unassigned'};

9. Since it has been used to differentiate multiple subsystems associated with single reactions in *fbamodel.subSystems*, the word 'and' can be used as a string delimiter to divide subsystem names across a cell array of separate strings:

% Replace existing instances of 'and' with '&' within single subsystems

old_names = {'Metabolism of terpenoids and polyketides','Metabolism of terpenoids and polyketides',

'Metabolism of cofactors and vitamins','Coenzymes and prosthetic groups','Glycan biosynthesis and

metabolism','Nucleotides and nucleic acids','Nucleotide Metabolism','Carbohydrate Metabolism','Energy

Metabolism'};

new_names = {'Metabolism of terpenoids & polyketides','Metabolism of terpenoids & polyketides','

Metabolism of cofactors & vitamins','Coenzymes & prosthetic groups','Glycan biosynthesis &

metabolism','Nucleotides & nucleic acids','Nucleotide metabolism','Carbohydrate metabolism',' Energy

metabolism'};

subsystems = replace(subsystems,old_names,new_names);

% Split multiple subsystems across reactions into a cell array of strings by using 'and' as a delimiter

new_subsystems = regexpi(subsystems,'and','split');

% Remove trailing spaces from the end of each string

new_subsystems = strtrim(new_subsystems);

% Remove any blank cells remaining in the subsystems array

new_subsystems{12}(2)=[];

new_subsystems{225}(2)=[];

% Replace subsystem names in the model

fbamodel.subSystems = new_subsystems;

**CRITICAL:** Parsing strings at the correct positions within single subsystems and removing any trailing spaces and blank cells after name replacement are essential to ensure consistency and match strings accurately within subsystem names during Pathway-level PCA (optional) and Pathway-level correlation analysis (optional).

### Preparation of transcriptomic data

**Timing: 2 weeks to 1 month**

The transcriptomic profiles utilized in this study originate from three studies conducted by Ludwig and Bryant (2011, 2012a,b) that sequenced RNA reads for *Synechococcus* sp. PCC 7002 cells grown under different conditions (detailed in Table 2). Following their generation via SOLiD™ sequencing, the study by Yang et al. (2015) describes how these data have been preprocessed prior to their inclusion in our protocol. The reads obtained from the NCBI Sequence Read Archive (SRA) were filtered to eliminate low-quality reads and aligned against the *Synechococcus* genome using Burrows-Wheeler Aligner (BWA) software. Following this, the sequences that did not map to the reference genome, those that were mapped to the rRNA-coding regions or those aligned to more than one region were eliminated. The remaining uniquely mapped genes were converted into reads per kilobase million (RPKM) and fold change values.

**Table 2:** Experimental conditions

Condition	Description of culture conditions	Reference
Standard control	Medium A+ at 38°C, illuminated at 250 μmol photons m^−2^s^−1^, sparged in air with 1% (v/v) CO_2_, with cells harvested at OD_730nm_ = 0.7.	(Ludwig and Bryant, 2011)
Dark oxic	Incubated in darkness prior to harvest, sparged in N_2_	(Ludwig and Bryant, 2011)
Dark anoxic	Incubated in darkness prior to harvest	(Ludwig and Bryant, 2011)
High light	Illuminated at 900 μmol photons m^−2^ s^−1^ prior to harvest	(Ludwig and Bryant, 2011)
OD 0.4	Harvested at OD_730nm_ = 0.4	(Ludwig and Bryant, 2011)
OD 1.0	Harvested at OD_730nm_ = 1.0	(Ludwig and Bryant, 2011)
OD 3.0	Harvested at OD_730nm_ = 3.0	(Ludwig and Bryant, 2011)
OD 5.0	Harvested at OD_730nm_ = 5.0	(Ludwig and Bryant, 2011)
Low O_2_	Sparged in N_2_	(Ludwig and Bryant, 2011)
Low CO_2_	Sparged with air [0.035% (v/v) CO_2_]	(Ludwig and Bryant, 2012a)
N-limited	Cells washed in medium A (lacking NO^3−^) and resuspended	(Ludwig and Bryant, 2012a)
S-limited	Cells washed with MgCl_2_	(Ludwig and Bryant, 2012a)
PO_4_^3-^ limited	Cells washed without (PO_4_^3-^) harvested at OD 730 nm = 0.7	(Ludwig and Bryant, 2012a)
Fe-limited	Cells washed in medium A with 720 μM deferoxamine me- sylate B added at OD_730nm_ = 0.35	(Ludwig and Bryant, 2012a)
NO_3_^-^	Standard growth in medium A (lacking NaNO_3_) with 25 mM HEPES, 1 μM NiSO_4_, 12 mM NaNO_3_	(Ludwig and Bryant, 2012a)
NH_3_	Standard growth in medium A (lacking NaNO_3_) with 25 mM HEPES, 1 μM NiSO_4_ and 10 mM NH_4_Cl	(Ludwig and Bryant, 2012a)
CO(NH_2_)_2_	Standard growth in medium A (lacking NaNO_3_) with 25 mM HEPES, 1 μM NiSO_4_ and 10 mM CO(NH_2_)_2_	(Ludwig and Bryant, 2012a)
Heat Shock	1 h heat shock at 47°C	(Ludwig and Bryant, 2012b)
22°C	Standard growth at 22°C	(Ludwig and Bryant, 2012b)
30°C	Standard growth at 30°C	(Ludwig and Bryant, 2012b)
Oxidative stress	5 μM methyl viologen added 30 min prior to harvesting	(Ludwig and Bryant, 2012b)
Mixotrophic	Medium A+ supplemented with 10 mM glycerol	(Ludwig and Bryant, 2012b)
Low salt	Medium A+ containing 3 mM NaCl and 0.08 mM KCl	(Ludwig and Bryant, 2012b)
High salt	Medium A+ containing 1.5 M NaCl and 40 mM KCl	(Ludwig and Bryant, 2012b)

Starting from RPKM values (stored in Datasets 1 and 2), we begin by recalculating fold changes as values centered around 1. As outlined in before you begin, this ensures a more convenient comparison between transcript and flux data when they are concatenated and also between all growth conditions, including the standard controls within each separate dataset, which were averaged over three replicates.

10. Download Dataset1.xls and Dataset2.xls from https://github.com/Angione-Lab/Synechococcus7002-metabolic-modelling/tree/master/transcriptomic_data.

**CRITICAL:** In this instance, all transcriptomic reads were obtained from studies conducted in tandem (with the same number of samples). For omic data obtained from multiple sources/studies that require additional normalization, see troubleshooting problem one.

11. Import the datasets into MATLAB:

% Import gene expression data from Excel

Dataset1 = readtable('Dataset1.xlsx');

Dataset2 = readtable('Dataset2.xlsx');

% Save columns containing RPKM values for each growth condition within numerical matrices

Dataset1RPKM = table2array(Dataset1(:,[3,4,5,6,10,14,18,22,26,30,34,38,42,46,50,54,58,62,66]));

Dataset2RPKM = table2array(Dataset2(:,[3,4,5,6,10,14,18,22,26,30]));

12. Within each dataset, divide the RPKM values for each experimental condition by the mean of three standard control values. This produces a series of fold change values centered around 1:

% Calculate separate standard averages for each dataset by computing the mean of three standard

control replicates (the first three columns of each RPKM matrix)

Standard_Averages_Dataset1 = mean(Dataset1RPKM(:,1:3),2);

Standard_Averages_Dataset2 = mean(Dataset2RPKM(:,1:3),2);

% Create dataset of transcript fold changes by dividing RPKM values in each growth condition

by the average RPKM value of standard control replicates in that dataset

newFC = Dataset1RPKM(:,4:19)./Standard_Averages_Dataset1;

newFC2 = Dataset2RPKM(:,4:10)./Standard_Averages_Dataset2;

transcripts = horzcat(newFC,newFC2);

13. Save fold change values for all growth conditions:

% Save names for each growth condition corresponding to the transcripts

conditions = {'Darkoxic','Darkanoxic','Highlight','OD04','OD10','OD30','OD50',

'lowO2','lowCO2','Nlim','Slim','Plim','Felim','Nitrate','Ammonia','Urea',

'Heatshock','T22','T30','Oxstress','Mixotrophic','Lowsalt','Highsalt'};

% Create a table by concatenating names of growth conditions and transcript fold changes

transcripts_table = array2table(transcripts,'VariableNames',conditions);

save('transcripts_table.mat','transcripts_table');

% Save as .mat variable and .csv file for later analyses

transcripts = transcripts'; % transpose matrix

save('transcripts.mat','transcripts');

writemat(transcripts,'transcriptsnew.csv');

## Key resources table

**Table:** 

REAGENT or RESOURCE	SOURCE	IDENTIFIER
**Deposited data**		

Sequence reads for *Synechococcus* sp. PCC 7002 cells	Ludwig and Bryant (2011)	Sequence Read Archive (SRA): https://www.ncbi.nlm.nih.gov/sra SRP004049
Sequence reads for *Synechococcus* sp. PCC 7002 cells	Ludwig and Bryant (2012a)	Sequence Read Archive (SRA): https://www.ncbi.nlm.nih.gov/sra SRP007372
Sequence reads for *Synechococcus* sp. PCC 7002 cells	Ludwig and Bryant (2012b))	Sequence Read Archive (SRA): https://www.ncbi.nlm.nih.gov/sra SRP013965
*Synechococcus*-metabolic-modeling Dataset 1	https://github.com/Angione-Lab/Synechococcus7002-metabolic-modelling/blob/master/transcriptomic_data/Dataset1.xlsx	N/A
*Synechococcus*-metabolic-modeling Dataset 2	https://github.com/Angione-Lab/Synechococcus7002-metabolic-modelling/blob/master/transcriptomic_data/Dataset2.xlsx	N/A

**Experimental models: organisms/strains**		

*Synechococcus* sp. PCC 7002 genome-scale model	Hendry et al. (2016)	N/A

**Software and algorithms**		

MATLAB R2020b	https://www.mathworks.com/products/matlab	N/A
Git 2.3.0	https://git-scm.com/	N/A
COBRA Toolbox v3.0	https://opencobra.github.io/cobratoolbox/stable/	N/A
Gurobi Optimizer 9.1.1	https://www.gurobi.com/	N/A
R-3.6.2 for Windows (64 bit)	https://cran.r-project.org/	N/A

**Other**		

Lenovo G50-30 80G0 model laptop computer (4 GB RAM, Intel Pentium 2.16 GHz processor and 500 GB solid-state hard drive)	Any reasonably up-to-date computer may be used	N/A

## Materials and equipment

Throughout this work, a Lenovo G50-30 80G0 model laptop computer using the Microsoft Windows 10 Home operating system was used. This computer has a 500 GB solid-state hard drive, an Intel Pentium N3530 CPU @ 2.16 GHz (1,333 Mhz memory speed and 4 cores) and 4 GB Random Access Memory (RAM). However, any reasonably up-to-date computer may be used to run all code and any operating system can be used - Windows, Mac OS, or Unix/Linux.

MATLAB (MathWorks: https://www.mathworks.com/products/matlab)

***Alternatives:*** While the current implementation applies the COBRA Toolbox in MATLAB, the package is extendable to any other platforms that support COBRA (such as Python, Julia, Mathematica as well as Linux, Windows and Mac binaries). A full list is available from: https://opencobra.github.io/.

Gurobi (https://www.gurobi.com/)

***Alternatives:*** While the current implementation uses the Gurobi Optimizer, a number of other available solvers could be installed and selected as the solver for quadratic optimization (such as IBM CPLEX https://www.ibm.com/products/ilog-cplex-optimization-studio, TOMLAB CPLEX https://tomopt.com/tomlab/download/products or MOSEK https://www.mosek.com/downloads).

## Step-by-step method details

**Figure 2:**
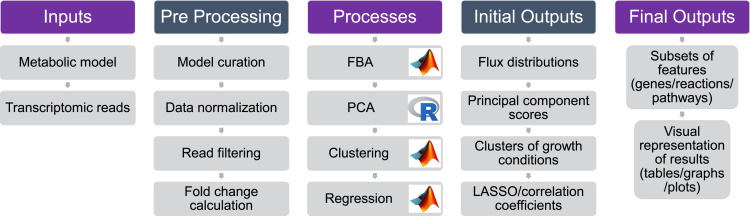
Inputs and outputs for all stages of the analysis in step-by-step method details.

In this section, a comprehensive step-by-step protocol is laid out for running the flux balance analysis of *Synechococcus* sp. PCC 7002, followed by principal components analysis, *k*-means clustering, LASSO regression and finally, correlation analysis. Each of these stages comprises a series of inputs and outputs, as well as intermediary processes that transform each type of data (see Figure 2). Critical steps for running the code and troubleshooting are interspersed between these steps and further elaborated in the troubleshooting section. All steps described in the code are case-specific, but they can easily be adapted to any transcriptomic dataset or GSMM that the user wishes to analyze.

**Figure 3:**
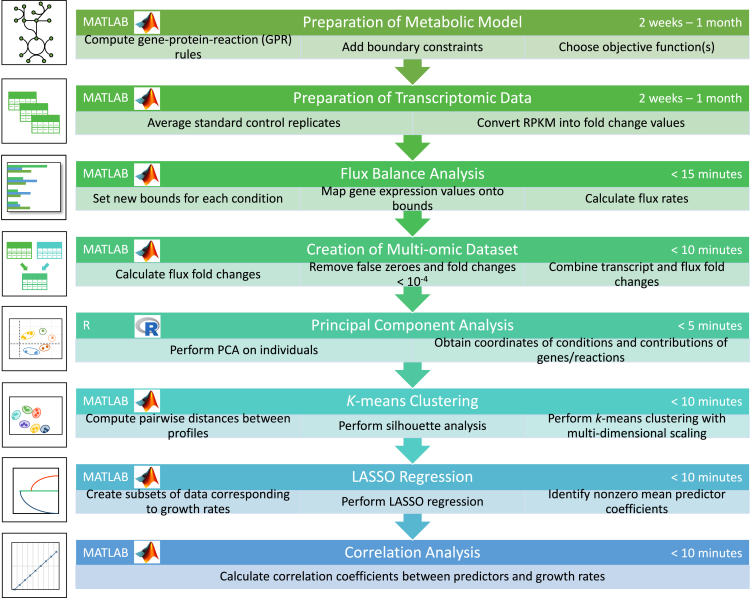
A workflow summarizing all stages of analysis in step-by-step method details.

### Flux balance analysis

**Timing: <15 min**

**Figure 4:**
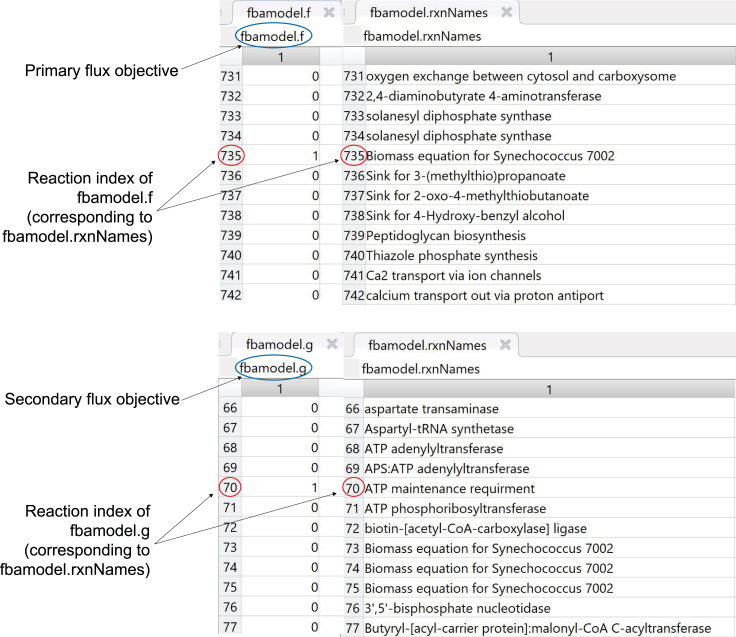
Check that the correct reaction indices for flux objectives *fbamodel.f* and *fbamodel.g* are selected in *fbamodel.mat* (indicated by the position of 1 in each vector).

***Note:*** During flux balance analysis, a single objective is usually specified for optimization within the field *fbamodel.c*. Using different solvers to perform the same optimization can cause solutions to vary, owing to differences in numerical implementation and the existence of multiple optimal solutions in the solution space. Calculating a unique solution using quadratic optimization is therefore more reliable when the flux distribution is intended for use in further analyses. To this end, minimizing the sum of squared flux values (L2 norm) carried by the metabolic network following maximization of the primary objective guarantees a unique set of flux solutions drawn from a strictly convex space (Angione, 2019). This section lists the major processes and steps for running a regularized flux balance analysis that maximizes pairwise objective functions in a bi-level fashion with a penalty term that considers the norm-2 of the flux vector (Heirendt et al., 2019). Bi-level regularized FBA is conducted in MATLAB using the quadratic programming solver Gurobi to compute flux distributions by selecting pairs of reactions in the GSMM to act as flux objectives (i.e. by selecting reactions within *fbamodel.f* and *fbamodel.g*, as detailed in Figure 4). Subsequently, 24 condition-specific growth profiles of *Synechococcus* sp. PCC 7002 are generated by integrating omics data relating to different environmental conditions, and three pairs of reactions are optimized for each of these profiles, namely: (i) Biomass - ATP maintenance (ii) Biomass - Photosystem I and (iii) Biomass - Photosystem II.

***Note:*** When calculating the flux distribution across conditions, the biomass reaction was chosen as the primary objective, while the secondary objective was set to ATP maintenance, photosystem I or photosystem II reactions in order to reflect the main cellular goals of cyanobacteria. In our case, the carbon-limited biomass reaction has been chosen as a primary objective to represent the maximization of growth rate and cellular yields (Feist and Palsson, 2010; Yuan et al., 2016; Lakshmanan et al., 2019), which is a critical consideration for cyanobacteria as this informs the substrate uptake rates and maintenance requirements that indicate fundamental cellular growth requirements. The chosen secondary objectives are key pathways involved in energy metabolism during photosynthesis. Simulating the cost of ATP maintenance helps to assess the energy required for sustaining metabolic activity even in the absence of growth. The incorporation of the photoexcitation reactions occurring within photosystems I and II serves to characterize how flux under various conditions reflects the light harvesting and energy transfer via photon absorption through these complexes. Thus, solving the quadratic optimization problem for multiple pairs of objectives helped to resolve trade-offs by considering the conditions and constraints affecting each of these objectives.

It has been established that the activity of biosynthetic and energy-generating pathways increases with the growth rate (Bernstein et al., 2014), which led us to implement multi-level regularized FBA in our pipeline, considering more than one objective function. This allows us to examine the effect of maximizing biomass using regularized flux balance analysis, followed by the maximization of flux through ATP maintenance and photosynthetic reactions. Performing the FBA in this manner has a relatively low computational cost, taking approximately 0.9–1.69 s per growth condition, and 43.53 s to run the entire script.

***Note:*** As an alternative to regularized FBA, we also provide a critical step detailing how users can employ flux variability analysis (FVA) to obtain minimal and maximal flux ranges for each growth condition. The full details for running the analysis are contained in the script *RUN_all.m* stored in the GitHub repository listed in the key resources table: https://github.com/Angione-Lab/Synechococcus7002-metabolic-modelling.

1. Firstly, we load the required variables within a local directory available to MATLAB:

% Load the pre-existing variables

% Genome-scale model ofSynechococcus sp. PCC 7002

load('SynechococcusPCC7002.mat');

% Array indexing the position of genes within reactions

load('pos_genes_in_react_expr.mat');

% Array defining the connection between genes and reactions based on GPR rules

load('reaction_expression.mat');

% Array indexing genes (required when replacing genes with their expression values)

load('ixs_genes_sorted_by_length.mat');

% List of gene IDs extracted from transcriptomic reads file

load('Syn7002_IDs.mat');

% Array of fold changes calculated from transcriptomic reads

load('transcripts.mat');

2. We then specify variables for the genes within the model and those included in the transcriptomic data:

% Create a variable to store gene accession IDs from the model

genes = fbamodel.genes;

% Create a variable to store gene accession IDs from the transcriptomic datasets

genes_in_dataset = Syn7002_IDs;

% Specify the number of objectives for FBA

M = 2;

% Specify the number of variables for FBA (i.e. genes)

V = numel(genes);

% Create indices to set the objective functions for FBA

ix_f = find(fbamodel.f==1); %check current primary objective

ix_g = find(fbamodel.g==1); %check current secondary objective

3. This step assigns indices for selecting the objective function(s) to be optimized during flux balance analysis:
  a. This step assigns indices for selecting the objective function(s) to be optimized during flux balance analysis.

% Set new primary objective f as the standard biomass reaction

ix_new_f = 735;

% Set new secondary objective g as ATP maintenance, photosystem I or photosystem II (manually change the

second objective optimized for FBA in each of the three cases by commenting out the other two objectives

not in use)

ix_new_g = find(ismember(fbamodel.rxnNames,'ATP maintenance requirment')==1);

% ix_new_g = find(ismember(fbamodel.rxnNames,'Photosystem I Reaction (cytochrome c6)')==1);

% ix_new_g = find(ismember(fbamodel.rxnNames,'photosystem II reaction')==1);

% Select new objective functions for simulation

fbamodel.f(ix_f) = 0;

fbamodel.f(ix_new_f) = 1;

fbamodel.g(ix_g) = 0;

fbamodel.g(ix_new_g) = 1;

**CRITICAL:** Although a large number of studies express the maximization of biomass as the only objective when performing FBA, it is important to recognize that, in reality, most organisms have multiple objectives to satisfy. Depending on the goal of the flux simulation, any reactions within the metabolic network reflecting a property of interest that must be optimized by the cell can be selected as objective functions via vector indexing. Within each pair of objectives, the primary flux objective *fbamodel.f* is fixed as the standard biomass reaction (*fbamodel.rxnNames* = 735) since it reflects the universal property of cellular growth maintenance, whereas the secondary flux objective *fbamodel.g* is manually switched between the reactions for ATP maintenance (*fbamodel.rxnNames* = 70), Photosystem I (*fbamodel.rxnNames* = 698) or Photosystem II (*fbamodel.rxnNames* = 697) to examine processes relating to energy metabolism and photosynthesis. As an alternative approach, users may also wish to force flux by increasing the lower bounds of reactions to ensure a minimum flux through pathways of interest, although in general this would not allow the user to find solutions that maximize their usage.

**CRITICAL:** Before applying gene-expression derived constraints during FBA, additional boundary constraints based on the varying metabolic capability of cells under different growth conditions (stored in *bounds.mat*) are used to modify lower and upper bounds in the model (*fbamodel.lb* and *fbamodel.ub*), thus shrinking the solution space and refining phenotypic prediction of metabolic activity. For all experimental conditions, a series of uptake and secretion rates are adjusted in the GSMM prior to performing FBA, taking into account: (i) composition of growth media limitation/supplementation of trace elements, e.g. nitrogen, sulfur, iron, phosphorus, etc. (ii) optical density at which cells were harvested (OD_730nm_ = 0.4/0.7/1.0/3.0/5.0), (iii) mode of energy utilization (phototrophy/heterotrophy/mixotrophy), (iv) availability of oxygen/carbon dioxide (low O_2_, low CO_2_, oxic/anoxic), (v) light intensity (dark or high light), (vi) temperature (22°C, 30°C, heat shock), (vii) salinity (low/high). This enables a more unique characterization of each growth condition.

***Note:*** For example, the bounds adjusted in our model are specified in Table 3, where a list of uptake and secretion rates (i.e. lower and upper bounds recorded in *fbamodel.lb* and *fbamodel.ub* respectively) for various exchange reactions are fixed at different values according to the growth conditions under which the *Synechococcus* cells were cultured and harvested (Ludwig and Bryant, 2011, 2012b,a). Apart from glycerol in the mixotrophic condition, lower bounds for other carbon sources (maltohexaose, maltopentaose, maltotriose, maltotetraose, maltose) and carbonate are set to zero for all conditions. γ represents the photon exchange reaction, whose lower bounds are determined using the calculation specified in Equation 1.

***Note:*** To specify the variation in light uptake across growth conditions, we calculated a photon uptake rate (P_U_) for each growth condition using a method similar to Vu et al. (2012). In this calculation, light consumption (LC) under each condition (mmol) is multiplied by the surface area (SA) of the culture exposed to the light source (m^2^); the product is subsequently divided by the total available dry cell weight (DCW) of the culture (grams per volume) as follows:

(Equation 1) $$P_{U}=\frac{LC\;\times SA}{DCW}$$

In this instance, the surface area of the culture exposed to the light source was calculated using the diameter of the cylindrical culture tube and the volume of the culture medium (Ludwig and Bryant, 2011), but users are advised to consider the shape and capacity of the vessel used to culture the cells in their own experimental setting when calculating this value.

***Note:*** If conducting growth experiments to directly measure light availability and DCW *in vivo* is not possible, users can refer to the literature to find the closest estimates available for their model species. In our case, we use an approximation for the DCW of marine *Synechococci* (Myers et al., 2013), which was confirmed to be in the same range of values as other *Synechococci* (Aikawa et al., 2014; Qiao et al., 2018). Upon obtaining these estimates or measured values, a linear calibration for cultures can be used to calculate the DCW from optical density (Kato et al., 2017), or a piecewise linear approximation can be adopted to extrapolate the line, calculate its gradient and obtain the growth rate.

4. Specify this series of boundary constraints to simulate growth media for each condition and record experimentally feasible growth rates:

% Load list of variables including reaction names, indices and new values for lower and upper bounds

in the model for each condition

load('bounds.mat');

5. In this step, Gurobi is specified as the solver to be used for FBA:

%% Solver

% Set Gurobi as the solver for linear and quadratic problems

changeCobraSolver('gurobi','LP');

changeCobraSolver('gurobi','QP');

% Avoid solver feasibility error

changeCobraSolverParams('QP', 'method', 1);

6. The new boundary constraints are assigned within *fbamodel.lb* and *fbamodel.ub* before running FBA in order to characterize condition-specific flux rates:

%% Set new bounds for standard control condition

fbamodel.lb(new_lb_ixs) = new_lb_val(1:15,1);

fbamodel.ub(new_ub_ixs) = new_ub_val(1:2,1);

7. Following this, a new vector of gene expression values (*x*) is mapped onto flux bounds for every condition, starting with an all-ones configuration for the standard control:

%% Flux distribution in standard control condition

% Set an all*−*one configuration for gene expression in the control condition

x = ones(numel(genes),1);

% Calculate flux rates for the control condition

[v1_control, f_out_control] =

evaluate_objective_minNorm(x,M,V,fbamodel,genes,reaction_expression,

pos_genes_in_react_expr,ixs_genes_sorted_by_length);

**CRITICAL:** Users could also use alternative methods for constraining the model using gene expression data. For a critical guide of factors to consider when integrating gene expression or other omic data with GSMMs, see troubleshooting problem two.

8. Alternatively, the function for flux balance analysis (*evaluate_objective_minNorm*) can be replaced by a function for flux variability analysis (*evaluate_objective_FVA*) to obtain minimal and maximal flux vectors:

% Calculate flux ranges for the control condition

[minFlux_control,maxFlux_control] =

evaluate_objective_FVA(x,M,V,fbamodel,genes,reaction_expression,pos_genes_in_react_expr,

ixs_genes_sorted_by_length);

**CRITICAL:** If using FVA instead of FBA, change the field *fbamodel.f* to *fbamodel.c* prior to calling *evaluate_objective_FVA* to ensure compatibility with the *fluxVariability.m* script, i.e. :

% Rename fbamodel.f as fbamodel.c if conducting FVA instead of FBA

if isfield(fbamodel, 'f')

 fbamodel.c = fbamodel.f;

end

9. All other conditions specify a loop to replace the RNA-seq expression. The dark oxic condition is provided as an example below:

%% Set new bounds for dark oxic condition

fbamodel.lb(new_lb_ixs) = new_lb_val(1:15,2);

fbamodel.ub(new_ub_ixs) = new_ub_val(1:2,2);

%% Flux distribution in dark oxic condition

% Choose growth condition by changing column vectors 1*−*23 in the transcripts dataset

expr_profile = transcripts(:,1);

pos_genes_in_dataset = zeros(numel(genes),1);

% Remove the last two characters (e.g. '.1') since transcripts are indicated with '.1' in the model but these are not present in the dataset

expression = '[.]\d';

replace = '';

genes_truncated = regexprep(genes,expression,replace);

% Set gene expression to the set of transcript fold changes in the selected growth condition

for i = 1:numel(genes)

 position = find(strcmp(genes_truncated{i},genes_in_dataset));

 if ∼isempty(position)

 pos_genes_in_dataset(i) = position;

 x(i) = expr_profile(pos_genes_in_dataset(i));

 end

end

% Specify the number of variables

V = numel(genes);

% Calculate flux rates for the dark oxic condition

[v1_do, f_out_do] =evaluate_objective_minNorm(x,M,V,fbamodel,genes,reaction_expression,

 pos_genes_in_react_expr,ixs_genes_sorted_by_length);

10. Similar to Step 8, the flux ranges for each condition can be calculated by replacing the *evaluate_objective_minNorm* with *evaluate_objective_FVA*:

% Calculate flux ranges for the dark oxic condition

[minFlux_do,maxFlux_do]=

 evaluate_objective_FVA(x,M,V,fbamodel,genes,reaction_expression,pos_genes_in_react_expr,

 ixs_genes_sorted_by_length);

**CRITICAL:** In Equation 2, we use the logarithmic vector-valued function φ to map the expression level of each gene set (represented by the vector θ) to a coefficient for the lower- and upper-limits of the corresponding reaction. Here, γ represents the “strength” of gene expression mapped to each reaction - which can be varied to adjust the level of upregulation or downregulation in cases where the values are too low to influence the flux rates (see troubleshooting problem two). This ensures higher metabolic sensitivity by enabling fine-tuning of flux rates by gene expression values to yield experimentally feasible fluxes for all growth conditions.

(Equation 2) $$\phi \left(\theta \right)={\left[1+\gamma |\log\left(\theta \right)\right]}^{sgn\left(\theta -1\right)}$$

11. For each condition, the function *evaluate_objective_minNorm* uses the instruction below to perform regularized flux balance analysis:

% This command is integrated within evaluate_objective_minNorm and does not need to be run separately

[solution] = optimizeCbModel(fbamodel,'max',1e*−*6);

f_out = solution.f;

v_out = solution.v;

12. If the function *evaluate_objective_FVA* is used in the place of *evaluate_objective_minNorm*, the instruction below gives norm-2 minimal and maximal flux vectors as outputs of flux variability analysis:

% This command is integrated within evaluate_objective_FVA and does not need to be run separately

[minFlux, maxFlux] = fluxVariability(fbamodel,[],[],[],0,1,'2-norm');

13. The same process is carried out for all growth conditions in the script until all resulting flux vectors can be concatenated within a single matrix:

% Concatenate flux vectors for all growth conditions

all_atp_flux = [v1_do,v1_da,v1_hl,v1_od04,v1_od10,v1_od30,v1_od50,v1_lo2,v1_lco2,v1_nlim,v1_slim,

 v1_plim,v1_felim,v1_no3,v1_nh3,v1_urea,v1_heat,v1_22c,v1_30c,v1_oxs,v1_mix,v1_ls,v1_hs, v1_control];

% Convert fluxes into absolute values, change all the values < 10ˆ*−*4 into 0 to account for

solver tolerance and save to a .csv file

all_atp_flux = abs(all_atp_flux)';

all_atp_flux(all_atp_flux <= 0.0001) = 0;

save('all_atp_flux.mat','all_atp_flux');

writematrix(all_atp_flux,'all_atp_flux.csv');

**CRITICAL:** In this case study, the threshold for setting flux values to zero was selected as 10^-4^, however we advise users of the protocol to choose their own cut-offs for flux values/fold changes by conducting a robustness analysis to assess different thresholds (see troubleshooting problem three).

**Figure 5:**
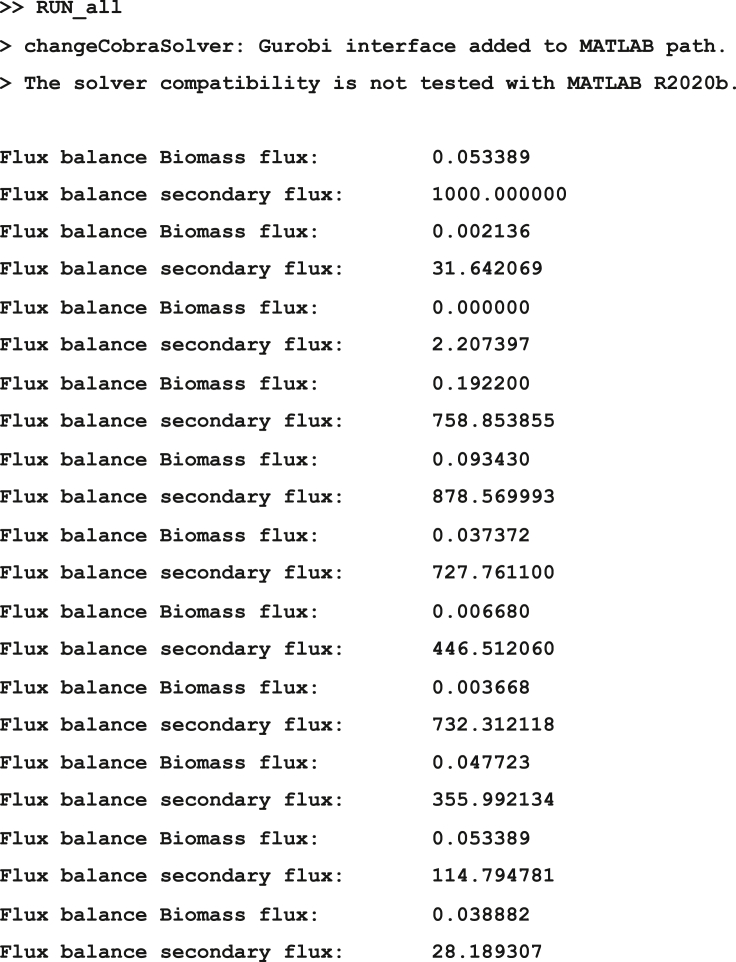
Example output of FBA when running the *RUN_all.m* script in MATLAB. The code prints flux values for the primary (biomass) and secondary flux objectives in all 24 growth conditions.

**Figure 6:**
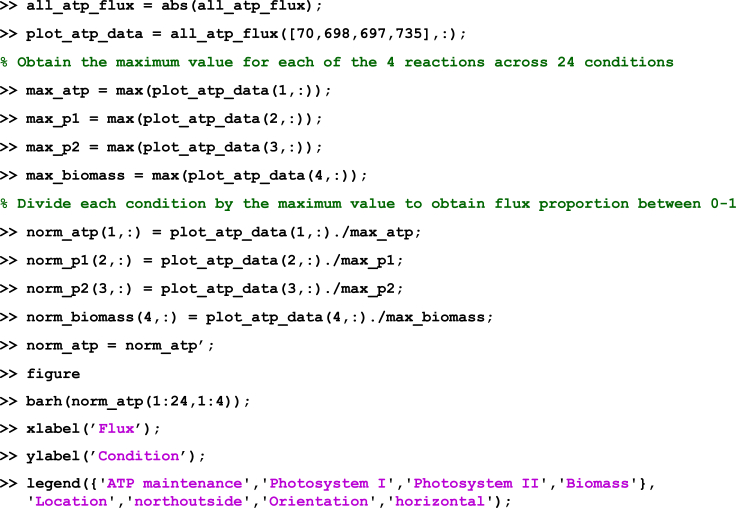
Plotting FBA results in the MATLAB console

**Figure 7:**
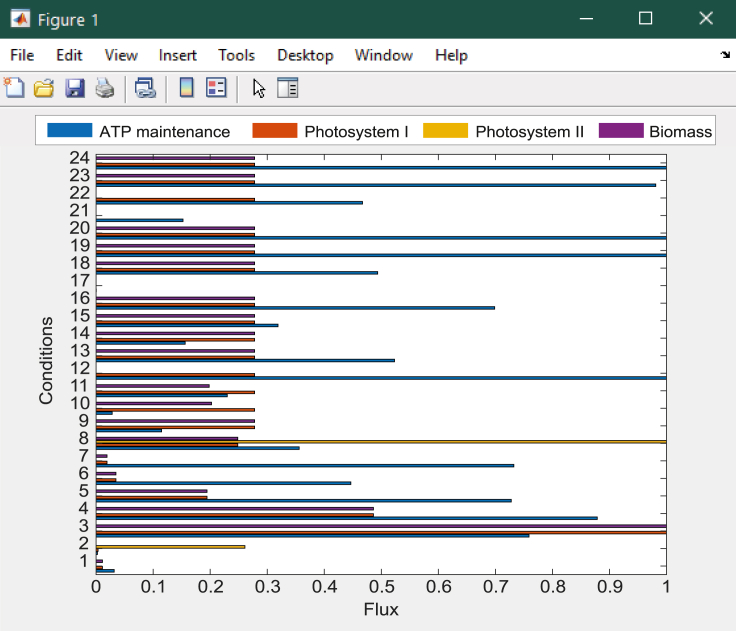
Example of horizontal bar chart plotted to display results of FBA for 4 key reactions. Flux rates in units of mmol/gDW h^-1^ have been re-scaled to values between 0-1 (see Figure 6 for plotting commands). Growth conditions are listed as follows: 1 - Dark oxic, 2 - Dark anoxic, 3 - High light, 4 - OD 0.4, 5 - OD 1.0, 6 - OD 3.0, 7 - OD 5.0, 8 - Low O_2_, 9 -Low CO_2_, 10 - N-limited, 11 - S-limited, 12 - PO_4_^3-^ limited, 13 - Fe-limited, 14 - NO_3_, 15 - NH_3_, 16 - CO(NH_2_)_2_, 17 - Heat Shock, 18°C - 22°C, 19°C - 30°C, 20 - Oxidative stress, 21 - Mixotrophic, 22 - Low salt, 23 - High salt, 24 - Standard Control. Further details of these experimental conditions are given in Table 2. Part of this figure is reprinted with permission from Vijayakumar et al. (2020).

**CRITICAL:** An example of the expected output for running the script *RUN_all.m* is provided in Figure 5. After flux rates have been calculated for all growth conditions, the results can be plotted as a simple bar chart where they are re-scaled as values between 0-1 (see Figure 6 for sample plotting commands and Figure 7 for the resulting plot).

**Table 3:** Flux bounds adjusted for FBA

fbamodel.lb										fbamodel.ub	
Condition	CO_2_	C_3_H_8_O_3_	SO_4_^2-^	NO_3_^-^	NH_4_^+^	CO(NH_2_)_2_	γ	O_2_	Fe^3+^	γ	O_2_
Standard control	−10	0	−1000	−1000	−1000	−1000	−0.065	−1000	−1000	1000	1000
Dark oxic	−10	0	−1000	−1000	−1000	−1000	−0.003	−1000	−1000	1000	1000
Dark anoxic	−10	0	−1000	−1000	−1000	−1000	−0.003	−0.01	−1000	1000	−0.01
High light	−10	0	−1000	−1000	−1000	−1000	−0.234	−1000	−1000	1000	1000
OD 0.4	−10	0	−1000	−1000	−1000	−1000	−0.114	−1000	−1000	1000	1000
OD 1.0	−10	0	−1000	−1000	−1000	−1000	−0.045	−1000	−1000	1000	1000
OD 3.0	−10	0	−1000	−1000	−1000	−1000	−0.008	−1000	−1000	1000	1000
OD 5.0	−10	0	−1000	−1000	−1000	−1000	−0.004	−1000	−1000	1000	1000
Low O_2_	−10	0	−1000	−1000	−1000	−1000	−0.065	−0.01	−1000	1000	−0.01
Low CO_2_	−0.01	0	−1000	−1000	−1000	−1000	−0.065	−1000	−1000	1000	1000
N-limited	−10	0	−1000	−0.01	−1000	−1000	−0.065	−1000	−1000	1000	1000
S-limited	−10	0	−0.01	−1000	−1000	−1000	−0.065	−1000	−1000	1000	1000
PO_4_^3-^ limited	−10	0	−1000	−1000	−1000	−1000	−0.065	−1000	−1000	1000	1000
Fe-limited	−10	0	−1000	−1000	−1000	−1000	−0.065	−1000	−0.01	1000	1000
NO_3_^-^	−10	0	−1000	−12	−1000	−1000	−0.065	−1000	−1000	1000	1000
NH_3_	−10	0	−1000	0	−10	−1000	−0.065	−1000	−1000	1000	1000
CO(NH_2_)_2_	−10	0	−1000	0	−1000	−10	−0.065	−1000	−1000	1000	1000
Heat Shock	−10	0	−1000	−1000	−1000	−1000	−0.065	−1000	−1000	1000	1000
22°C	−10	0	−1000	−1000	−1000	−1000	−0.065	−1000	−1000	1000	1000
30°C	−10	0	−1000	−1000	−1000	−1000	−0.065	−1000	−1000	1000	1000
Oxidative stress	−10	0	−1000	−1000	−1000	−1000	−0.065	−1000	−1000	1000	1000
Mixotrophic	−10	-10	−1000	−1000	−1000	−1000	−0.065	−1000	−1000	1000	1000
Low salt	−10	0	−1000	−1000	−1000	−1000	−0.065	−1000	−1000	1000	1000
High salt	−10	0	−1000	−1000	−1000	−1000	−0.065	−1000	−1000	1000	1000

**CRITICAL:** If calculating flux ranges, the minimum and maximum flux vectors can be used as two sets of fluxomic features, or users could calculate the mean flux between these two values for use in the next steps of the pipeline.

14. For flux variability analysis, the mean of minimal and maximal flux vectors for different conditions can be calculated as follows:

% Concatenate minimal and maximal flux vectors for all growth conditions

all_atp_minFlux =

 [minFlux_do,minFlux_da,minFlux_hl,minFlux_od04,minFlux_od10,minFlux_od30,minFlux_od50,minFlux_lo2,

 minFlux_lco2,minFlux_nlim,minFlux_slim,minFlux_plim,minFlux_felim,minFlux_no3,minFlux_nh3,minFlux_urea,

 minFlux_heat,minFlux_22c,minFlux_30c,minFlux_oxs,minFlux_mix,minFlux_ls,minFlux_hs,minFlux_control];

all_atp_maxFlux =

 [maxFlux_do,maxFlux_da,maxFlux_hl,maxFlux_od04,maxFlux_od10,maxFlux_od30,maxFlux_od50,maxFlux_lo2,

 maxFlux_lco2,maxFlux_nlim,maxFlux_slim,maxFlux_plim,maxFlux_felim,maxFlux_no3,maxFlux_nh3,maxFlux_urea,

 maxFlux_heat,maxFlux_22c,maxFlux_30c,maxFlux_oxs,maxFlux_mix,maxFlux_ls,maxFlux_hs,maxFlux_control];

% Calculate mean fluxes between minFlux and maxFlux ranges for each condition

all_atp_meanFlux = zeros(742,24);

for m = 1:24

 all_atp_meanFlux(:,m) = (all_atp_minFlux(:,m) + all_atp_maxFlux(:,m))./2;

end

### Creation of multi-omic dataset

**Timing: < 10 min**

In our analyses, gene transcripts constitute a vital component of the flux balance analysis since transcriptomic data are integrated into the GSMM to determine condition-specific flux values. Although partially based on transcriptomics, flux rates are additionally subjected to condition-specific GSMM constraints, the steady-state, and their underlying biochemistry. This automatically creates a component of nonredundant information that does not exist in the transcriptomic dataset. Generating flux data supplies more layers of information to further refine phenotypic predictions. It is thus easier to identify important predictors during machine learning analyses; much of the noise in the gene transcript data is no longer present in the flux data, since gene transcripts with low expression have been ‘filtered out’ as they do not have a large influence on linear constraints in the metabolic model, and consequently they have a smaller effect on the flux rates.

Therefore, if a machine learning model can extract the non-redundant information contained in the flux rates, they can contribute new mechanistic information that is not found in the transcriptomic data. Furthermore, the model itself can act as a tool for ranking and noise reduction since the effect of low importance genes can be 'filtered out' even if their expression is highly variable across conditions. Without the metabolic model, the importance of these genes would be overstated, and they would be used erroneously to differentiate conditions. For example, in our case study, reactions involved in succinate dehydrogenation (SUCD1Itlm/SUCD1Icpm), efflux (SUCCt2b) or exchange (EX_succ_E) were found to be positively correlated with growth for all three objective pairs and were also identified among the highest positive correlations when analyzing the concatenated dataset of gene transcripts and Biomass - ATP maintenance flux data (Vijayakumar et al., 2020). These reactions are encoded by A1094 and A2569, which had relatively low gene expression and variability across growth conditions (ranging between 0.33 to 3.74 and 0.14 to 3.66, respectively). Being unrelated to genes already identified as significant during LASSO and correlation analyses of the single omic (transcriptomic) data, these reactions were only detected as a result of transcriptomic data being used to adjust the constraints for calculating flux rates, showing the importance of the metabolic model in characterizing the phenotype across conditions.

In practice, combining transcript and flux data in a single multi-omic dataset (by converting them into fold change values) provides a direct point of comparison between the two omics and an opportunity to observe in which instances the flux values are more predictive than transcript values. Generally, transcriptomic and fluxomic data produce different outcomes from the modeling and statistical analyses and combining the two omics yields more stable predictions.

In this section, we define how to concatenate transcript and flux data by obtaining fold changes that enable a comparison of their contribution to gene/reaction variables as a result of the conditions under which the cells were grown and harvested.

15. In MATLAB, create datasets for further analysis by concatenating transcripts and fluxes:

% Find out the highest flux value in the fold change matrix by setting Inf values to 0 and omitting NaN values

ATP_FC_noinf = (all_atp_flux(1:23,:))./(all_atp_flux(24,:));

ATP_FC_noinf(isinf(ATP_FC_noinf)) = 0;

max_ATP_FC = max(ATP_FC_noinf,[],'all','omitnan');

% Divide flux values in all conditions by the standard control to obtain fold changes

ATP_FC = (all_atp_flux(1:23,:))./(all_atp_flux(24,:));

% Set all fold changes < 10^*−*4 equal to 0 to account for solver tolerance

ATP_FC(ATP_FC<=0.0001) = 0;

% Set all NaN values to 1

ATP_FC(isnan(ATP_FC)) = 1;

% Set Inf values equal to the highest flux value in the matrix

ATP_FC(isinf(ATP_FC)) = max_ATP_FC;

% Concatenate transcripts and flux fold changes

ATPTF = horzcat(transcripts,ATP_FC);

% Add a row of all ones to represent the fold change for the standard control

ATPTF(24,:) = ones;

% Save as .mat variable and .csv file for later analyses

save('ATPTF.mat','ATPTF');

writemat(ATPTF,'all_ATPTF.csv');

### Principal component analysis (PCA)

**Timing: < 5 min**

Principal component analysis (PCA) can reduce multidimensional datasets to a few latent dimensions known as principal components, allowing the identification of variables responsible for the largest variations within datasets. The reduction of dimensionality within voluminous omic datasets is an important process to achieve successful multi-omic integration and is vital to facilitate their interpretation.

In this analysis, PCA is being used to compare the contribution of each growth condition to the construction of dimensions that summarize the greatest proportion of variance in the dataset. Furthermore, specific genes and reactions contributing to variance between conditions can be pinpointed using Pathway-level PCA, wherein they are classified according to their genetic/metabolic function. The role of these genes and reactions in significant pathways or cellular processes can also be ascertained in a more detailed manner.

Here, principal component analysis is conducted in R using the FactoMineR and factoextra packages. Full details of the code are provided in the script *PCA_script.R*, which can be found in the GitHub repository listed in the key resources table: https://github.com/Angione-Lab/Synechococcus7002-metabolic-modelling. For users wishing to carry out the full analysis on gene transcripts and/or flux rates in the form of .mat variables in MATLAB, the function *pca* can be used to carry out PCA on raw data, *pcares* returns the residuals obtained by retaining a given number of principal components and *pcacov* performs PCA on the square covariance matrix. However, we demonstrate our pipeline using the packages in R for improved analysis and visualization of plots that facilitate the biological interpretation. As seen below, the R packages generate detailed plots, lists of variable contributions, principal component scores and the proportions of variance explained by each dimension.

**Figure 8:**
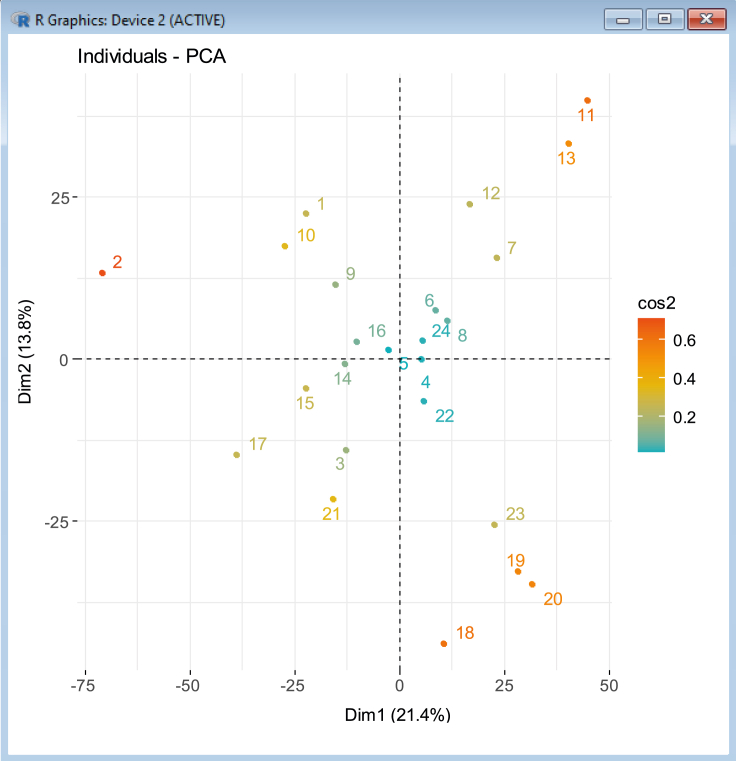
Example of principal component analysis plot of growth conditions colored according to cos2 values. The higher the cos2 value, the greater the proportion of contribution to the total distance, signifying greater importance of the principal components for that condition. Part of this figure is reprinted with permission from Vijayakumar et al. (2020).

**Figure 9:**
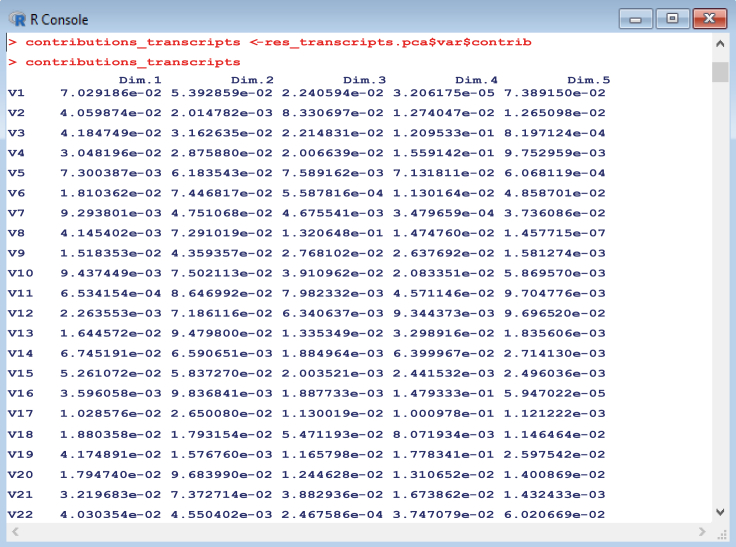
Obtaining principal component contributions for all variables (gene transcripts) in the dataset.

**Figure 10:**
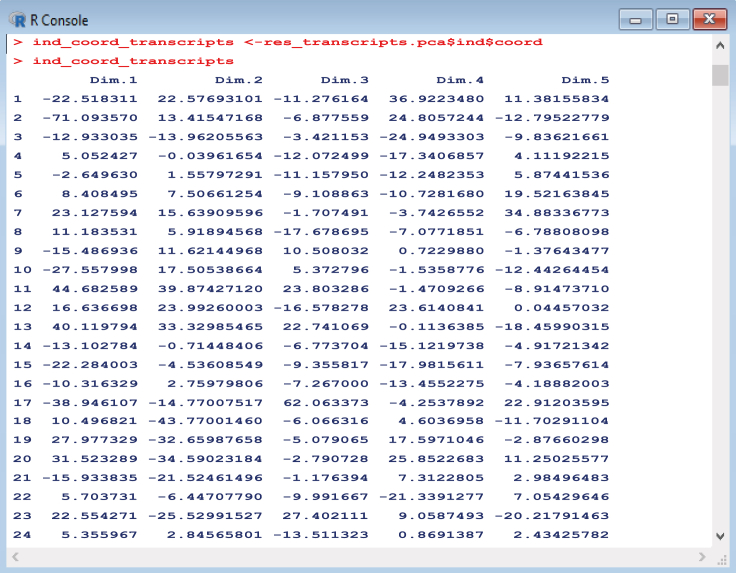
Obtaining coordinates for principal components according to individuals (growth conditions).

The gene transcripts dataset is used as an example below, but the same steps can be repeated for all datasets (*transcripts*, *all_ATP_flux*, *all_ATPTF*, etc.). For an example plot using individual growth conditions, see Figure 8. Other useful outputs resulting from the analysis, such as principal component contributions (Figure 9) or coordinates (Figure 10) relating to all growth conditions or variables within the dataset can also be saved for further inspection.

16. We begin by navigating to the workspace in R and loading the required packages:

setwd(C:/Users/)

library(devtools)

library(FactoMineR)

library(factoextra)

library(corrplot)

library(PerformanceAnalytics)

17. We then load transcript/multiomic/flux .csv data files for analysis:

transcripts <- read.csv(file = transcriptsnew.csv , head = FALSE,sep =,)

18. Perform PCA for each dataset:

res_transcripts.pca <- PCA(transcripts)

19. Create plots to compare principal components scores for the first two dimensions:

transcripts_PCA_plot <- fviz_pca_ind(res_transcripts.pca, col.ind = cos2,

gradient.cols = c(#00AFBB, #E7B800, #FC4E07),

repel = TRUE % Avoid text overlapping

)

***Note:*** The number of dimensions to be plotted can be adjusted, usually depending on the proportion of variance explained by each component. For each dataset, conditions are colored according to cos2 values that indicate the contribution of the first two components to the squared distance of each condition to the origin.

20. Obtain contributions of principal component variables (genes) for each dataset:

contributions_transcripts <- res_transcripts.pca$var$contrib

21. Obtain principal component coordinates for individual growth conditions:

ind_coord_transcripts <- res_transcripts.pca$ind$coord

### Pathway-level PCA

**Timing: < 15 min**

In order to carry out a more detailed investigation of specific gene transcripts or metabolic reactions in the model, it is possible to perform a pathway-level PCA that categorizes genes and reactions identified during PCA according to their main biological function. Upon obtaining the results of these analyses, we can plot the sum and average principal component contributions across different pathways as well as principal component coordinates for each growth condition against single reaction fluxes. As in the previous principal component analysis section, there are existing functions for plotting these data in MATLAB. The *barh* function can be used to generate bar plots displaying sums of subsystem contributions, the *polarplot* function can be used to display average contributions by subsystem and the *scatter* function can be used to plot principal coordinates for individual reactions against their corresponding flux values across different growth conditions. In this protocol, we utilize the *plotrix* and *fmsb* libraries in R to customize individual pyramid plots and radar charts, facilitating comparisons between different pairs of flux objectives and multiple pathways.

This provides an opportunity to study these components in a more detailed manner through expanding the scope of biological insights detected and establishing connections between genes and reactions within the same functional category or pathway. It is important to account for the varying number of reactions within each pathway, therefore both the sum and average contributions to variance can be used as measures of comparison from principal components. Additionally, principal component coordinates for each growth condition can also be compared against single reactions selected from the top flux contributors to variance (identified for all three objective pairs during Principal Component Analysis (PCA)). This helps to quantify the strength of association between these reactions and the principal components they are best summarized by.

22. Within MATLAB, import the table of contributions for the dataset (*all_atp_flux* is provided as an example):

% Import data table of flux contributions

contrib_ATP = readtable('contrib_all_atp_flux.csv');

% Concatenate with reaction and subsystem names from the GSMM

contrib_ATP_new =

horzcat(contrib_ATP(:,{'Var1'}),fbamodel.rxns,fbamodel.rxnNames,fbamodel.subSystems,contrib_ATP(:,{'Dim_1'

'Dim_2' 'Dim_3' 'Dim_4' 'Dim_5'}));

% Sort contributions in descending order by Dim1 then Dim 2

contrib_ATP_sort = sortrows(contrib_ATP_new,{'Dim_1','Dim_2','Dim_3','Dim_4','Dim_5'},{'descend' 'descend'

'descend' 'descend' 'descend'});

contrib_ATP_Dim1 = sortrows(contrib_ATP_new,{'Dim_1'},{'descend'});

contrib_ATP_Dim2 = sortrows(contrib_ATP_new,{'Dim_2'},{'descend'});

% Save vector containing flux contributions for the first and second dimensions, specifying a dataset of contributions

Dim_1_and_2 = table2array(contrib_ATP_new(:,5:6));

% Save all contributions to .xls file

writetable(contrib_ATP_sort,'contrib_atp_sort.xlsx');

writetable(contrib_ATP_Dim1,'contrib_atp_dim1.csv');

writetable(contrib_ATP_Dim2,'contrib_atp_dim2.csv');

***Note:*** While gene transcripts can be classified by their Cluster of Orthologous Genes (COG) category, reactions must be classified according to the pathways they are assigned within *fbamodel.subSystems*. Since each reaction can be classified by multiple subsystems, separate cell arrays can be allocated to store subsystems from each column of *fbamodel.subSystems*. The number of arrays needed depends on the maximum number of subsystems that a single reaction is categorized by within the model. In this case, each reaction is assigned to a maximum of five subsystems, therefore a total of five cell arrays are required to store the subsystem names, which are later concatenated into a single array and used to replace the original *fbamodel.subSystems* in the model.

23. Create cell arrays to store subsystems from *fbamodel.subSystems*:

% List all subsystems in the model

list_subsystems = unique([new_subsystems{:}])';

% Create cell arrays to store subsystem names

first_subsystems = cell(numel(list_subsystems),1);

...

fifth_subsystems = cell(numel(list_subsystems),1);

24. Write a 'for' loop to obtain the names of subsystems according to the number of subsystems that each reaction is categorized by:

for k = 1 : length(fbamodel.subSystems)

 thisCellContents = fbamodel.subSystems{k};

% Get the first subsystem for all reactions

 first_subsystems{k} = thisCellContents{1};

 if length(thisCellContents) > 1

% Get the second subsystem if present

 second_subsystems{k} = thisCellContents{2};

 else

% If there is only one subsystem for the reaction, assign the second a blank []

 second_subsystems{k} = [];

 end

 ...

 if length(thisCellContents) > 4

% Get the fifth subsystem if present

 fifth_subsystems{k} = thisCellContents{5};

 else

% If there are no more than four subsystems for the reaction, assign the fifth a blank []

 fifth_subsystems{k} = [];

 end

end

25. Create another series of cell arrays to store reaction indices; then retrieve the indices that match the number of subsystems (between one and five) for each unique subsystem:

% Specify the number of unique subsystems

N = length(list_subsystems);

% Create empty cell arrays (with length of list_subsystems) to store reaction indices of

each number of subsystems

ix_first = cell(N,1);

...

ix_fifth = cell(N,1);

% Retrieve reaction indices for each group of subsystems (1*−*5):

for s = 1:N

 ix_first{s} = find(strcmpi(list_subsystems{s},first_subsystems));

 ...

 ix_fifth{s} = find(strcmpi(list_subsystems{s},fifth_subsystems));

end

26. Merge all five arrays into a single list of indices for all subsystems:

% Concatenate all five columns

ix_all = horzcat(ix_first,ix_second,ix_third,ix_fourth,ix_fifth);

% Create cell array to store reaction indices for all subsystems

ixs_subsystems = cell(length(ix_all),1);

% Merge columns to compile a total list of indices for each subsystem

for a = 1:length(ixs_subsystems)

 ixs_subsystems{a} = vertcat(ix_all{a,:});

end

27. Create new variables to store the number of reactions as well as the sums and averages of principal component contributions:

% Create empty vector to store number of reactions within each pathway

cardinality_subsystems = zeros(numel(list_subsystems),1);

% Create empty vectors to store sums of contributions within each pathway for the first and second principal

components

sum_contrib_subsystems_PC1 = zeros(numel(list_subsystems),1);

sum_contrib_subsystems_PC2 = zeros(numel(list_subsystems),1);

% Create empty vectors to store average contributions within each pathway for the first and

second principal components

avg_contrib_subsystems_PC1 = zeros(numel(list_subsystems),1);

avg_contrib_subsystems_PC2 = zeros(numel(list_subsystems),1);

28. Calculate the sums and averages of flux contributions according to their respective subsystems using another 'for' loop:

%% Sort flux contributions according to subsystems

for i = 1:numel(list_subsystems)

% Compute the sums of contributions for the first and second principal components

 sum_contrib_subsystems_PC1(i) = sum(Dim_1_and_2(ixs_subsystems{i},1));

 sum_contrib_subsystems_PC2(i) = sum(Dim_1_and_2(ixs_subsystems{i},2));

% Record the number of reactions within each subsystem

 cardinality_subsystems(i) = numel(ixs_subsystems{i});

% Compute the mean contributions by dividing sums by the number of reactions

in each subsystem

 avg_contrib_subsystems_PC1(i) = sum_contrib_subsystems_PC1(i)./cardinality_subsystems(i);

 avg_contrib_subsystems_PC2(i) = sum_contrib_subsystems_PC2(i)./cardinality_subsystems(i);

end

29. Create a table containing all sums and averages of component contributions:

subsystem_names = array2table(list_subsystems,'VariableNames',{'Subsystems'});

subsys_sum_avg_ATP = horzcat(sum_contrib_subsystems_PC1,avg_contrib_subsystems_PC1,

sum_contrib_subsystems_PC2,avg_contrib_subsystems_PC2);

subsys_sum_avg_ATP_table = array2table(subsys_sum_avg_ATP,'VariableNames',{'PC1 Sum','PC1 Average',

'PC2 Sum','PC2 Average'});

subsys_sum_avg_ATP_table = horzcat(subsystem_names,subsys_sum_avg_ATP_table);

writetable(subsys_sum_avg_ATP_table,'pathway_contrib_ATP.csv');

***Note:*** Within this loop, *sum_contrib_subsystems* and *avg_contrib_subsystems* can be manually adjusted to select each dataset of contributions individually, i.e. *Dim_1_&_2* originating from *contrib_ATP_new*, *contrib_p1_new* or *contrib_p2_new*.

**Figure 11:**
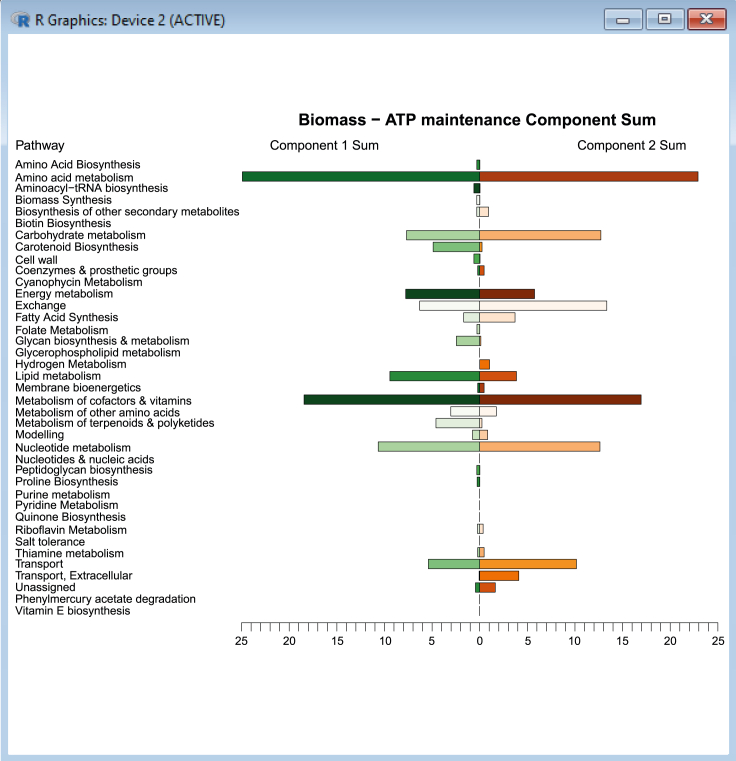
Sums of first and second principal component contributions across metabolic pathways (model subsystems) for the Biomass - ATP maintenance flux objective pair. Part of this figure is reprinted with permission from Vijayakumar et al. (2020).

30. The sums of contributions to variance within each subsystem can be summarized using a pyramid plot in R (Figure 11) to compare results between the first and second principal components:

# Load plotrix library

library(plotrix)

# Load all pathway contribution data

pathway_contributions <- read.csv(file = pathway_contrib_ATP. csv, head = TRUE, sep = ,)

# Load pathway labels in reverse order for plotting

pathways <- rev(pathway_contributions[,c(Subsystems)])

# Load pathway sums of contributions for Component 1 and Component 2 in reverse order

comp1atp.pop <- rev(pathway_contributions[,c(PC1.Sum)])

comp2atp.pop <- rev(pathway_contributions[,c(PC2.Sum)])

#Set ATP color gradient using preset-color-palettes from R-colorspace

library(colorspace)

comp1atpcol <- sequential_hcl(9,Greens)

comp2atpcol <- sequential_hcl(9,Oranges)

# Plot ATP pyramid

par(mar = pyramid.plot(comp1atp.pop, comp2atp.pop, labels = pathways, main = Biomass – ATP

maintenance Component Sum, top.labels = c(Component 1 Sum, Pathway,Component 2 Sum), unit = ,

lxcol = comp1atpcol, rxcol = comp2atpcol, gap = 0, xlim = c(25,25), show.values = FALSE))

**Figure 12:**
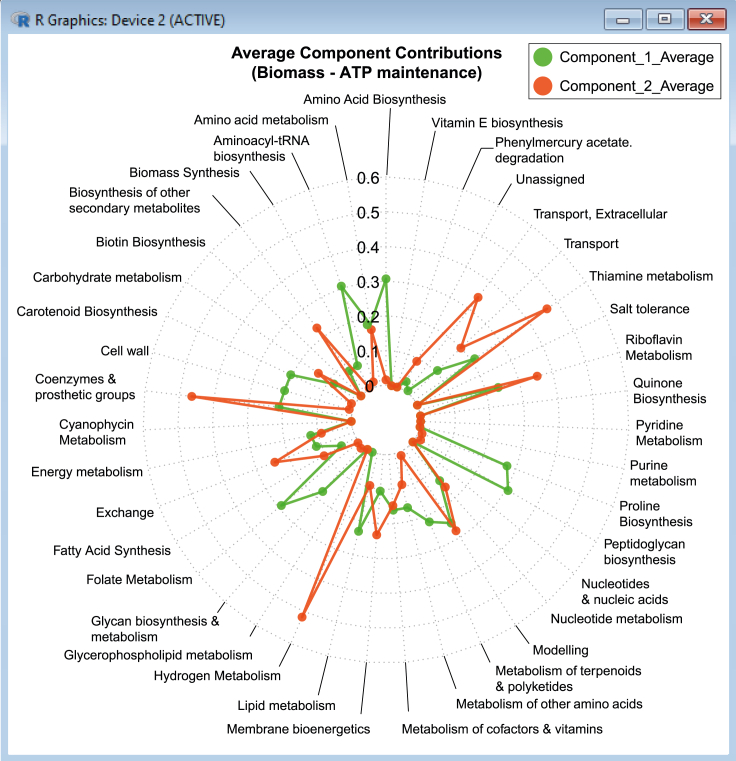
Averages of first and second principal component contributions across metabolic pathways (model subsystems) for the Biomass - ATP maintenance flux objective pair. Part of this figure is reprinted with permission from Vijayakumar et al. (2020).

31. Likewise, the average contributions to variance within each subsystem can be summarized using a radar chart in R (Figure 12) to compare results between the first and second principal components:

# Load fmsb library

library(fmsb)

# Load pathway names

pathways <- pathway_contributions [,c(Subsystems)]

# Load pathway average contributions for Component 1 and Component 2

PC1_Average <- pathway_contributions [,c(PC1.Average)]

PC2_Average <- pathway_contributions [,c(PC2.Average)]

# Specify the maximum and minimum values for plotting

max <- rep (c(0.6), each = 39)

min <- rep (c(0), each = 39)

# Create a dataframe of contribution values

ATP_radar_data <- t(data.frame(max,min,PC1_Average,PC2_Average))

# Specify labels for each data series

rownames(ATP_radar_data) = c(max,min,Component_1_Average,Component_2_Average)

colnames(ATP_radar_data) = pathways

# Convert the variable back into a data frame

ATP_radar_data <- data.frame (ATP_radar_data)

# Define line colors

colors_line_ATP <- c(scales :: alpha(green3,0.9),scales :: alpha(orangered,0.9))

# Create the plot (specifying the number of axis segments, title, line colors,axis labels, etc.)

radarchart (ATP_radar_data,

seg = 6,

title = Average Component Contributions (Biomass - ATP maintenance),

pcol = colors_line_ATP ,

plty = 1:1,

plwd = 2,

axistype = 4,

caxislabels = c(0,0.1,0.2,0.3,0.4,0.5,0.6),

cglty = 3,

cglcol = gray70,

axislabcol = gray0)

# Add a legend to indicate which series belongs to which component

legend (x = 1.35, y = 1.25, legend = rownames(ATP_radar_data [- c(1,2),]),bty = o,

pch = 20, col = colors_line_ATP, text.col = gray0, cex = 1.2, pt.cex = 3)

**Figure 13:**
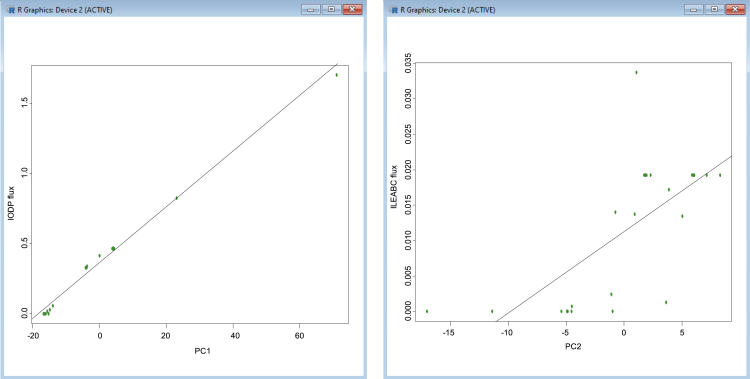
Example of principal component plots between principal component coordinates (*x*) and Biomass - ATP maintenance flux (*y*) across 24 growth conditions. Part of this figure is reprinted with permission from Vijayakumar et al. (2020).

***Note:*** Finally, we can also analyze principal component coordinates for each growth condition against single reaction fluxes. An example is demonstrated below using Biomass - ATP maintenance flux data in R (with the expected results plotted in Figure 13).

32. We begin by loading the requisite variables:

# Load all principal component coordinates

ind _ coord _ ATP <- read . csv ( file = ind _ coord _ all _ atp _ flux .csv, head = TRUE , sep = ,)

# Load all flux data and contributions sorted by PC1 and PC2

ATPflux <- read . csv ( file = all _ atp _ flux . csv, head = FALSE , sep = ,)

contrib _ ATP _ Dim1 <- read . csv ( file = contrib _ atp _ dim1 . csv, head = TRUE , sep = ,)

contrib _ ATP _ Dim2 <- read . csv ( file = contrib _ atp _ dim2 . csv, head = TRUE , sep = ,)

33. Select only the columns required

# Select the first principal component

PC1 _ ATP <- ind _ coord _ ATP [, c(Dim.1)]

# Check the reaction name and index of the highest contributor to the first principal component

head ( contrib _ ATP _ Dim1 )

# Select the flux rate corresponding to the reaction yielding the top

contribution in the first principal component

IODP <- ATPflux [, c (708)]

# Select the second principal component and the reaction corresponding to the

top contribution in the second principal component

PC2 _ ATP <- ind _ coord _ ATP [, c(Dim.2)]

# Check the reaction name and index of the highest contributor to the second principal component

head ( contrib _ ATP _ Dim2 )

# Select the flux rate corresponding to the reaction yielding the top

contribution in the second principal component

ILEABC <- ATPflux [, c (301)]

34. Use the data to fit linear models and create scatter plots for both principal components:

# Fit linear models

require (stats)

fit_ATP1 <- lm(IODP ∼ PC1_ATP)

fit_ATP2 <- lm(ILEABC ∼ PC2_ATP)

# Create plots

ATP1_plot <- plot (PC1_ATP, IODP , xlab = PC1, ylab = IODP flux, pch = 19 , col = chartreuse4, axes = TRUE)

ATP2_plot <- plot (PC2_ATP, ILEABC , xlab = PC2, ylab = ILEABC flux, pch = 19 , col = chartreuse4, axes = TRUE)

# Calculate the Pearson correlation coefficient

corr_PC1 = cor(PC1_ATP, IODP)

corr_PC2 = cor(PC2_ATP, ILEABC)

abline(fit_ATP1)

abline(fit_ATP2)

### *K*-means clustering

**Timing: < 10 min**

**Figure 14:**
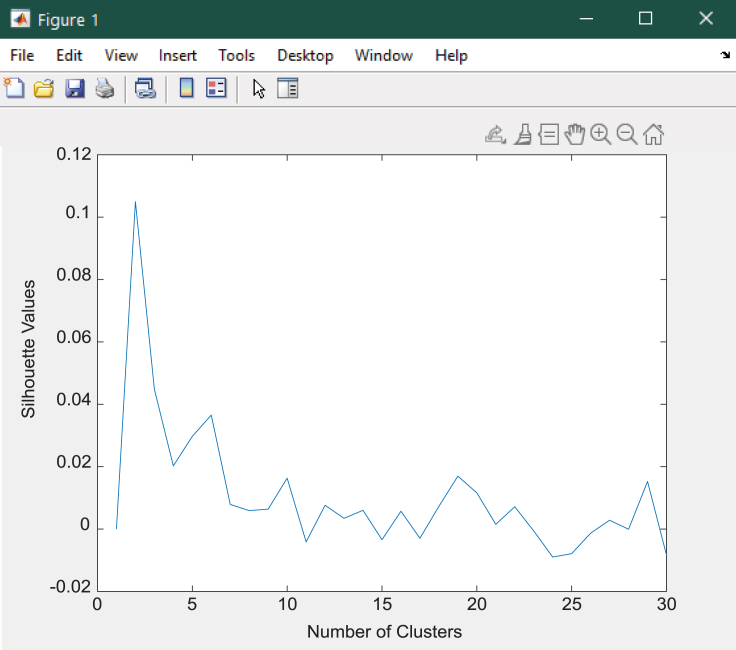
Example of silhouette pre-plot to determine the number of clusters to be used for *k*-means.

**Figure 15:**
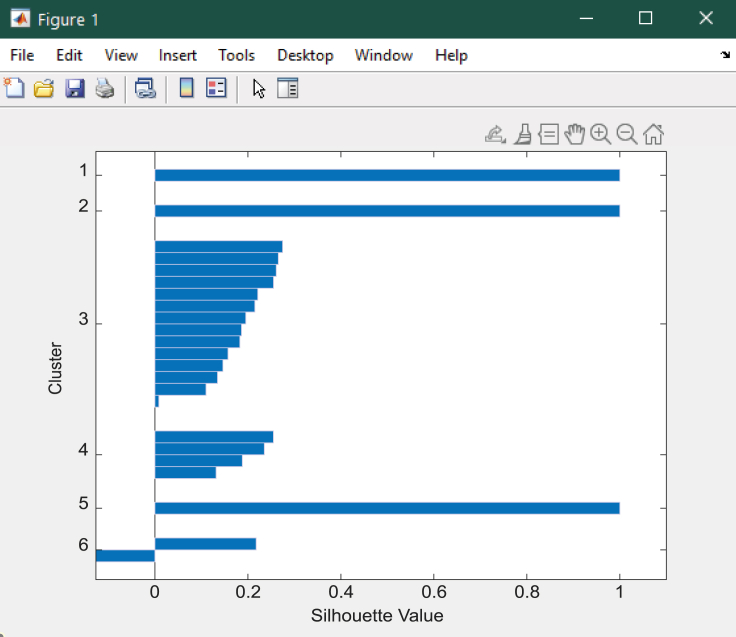
Example of silhouette plot for transcript data (*k*=6).

**Figure 16:**
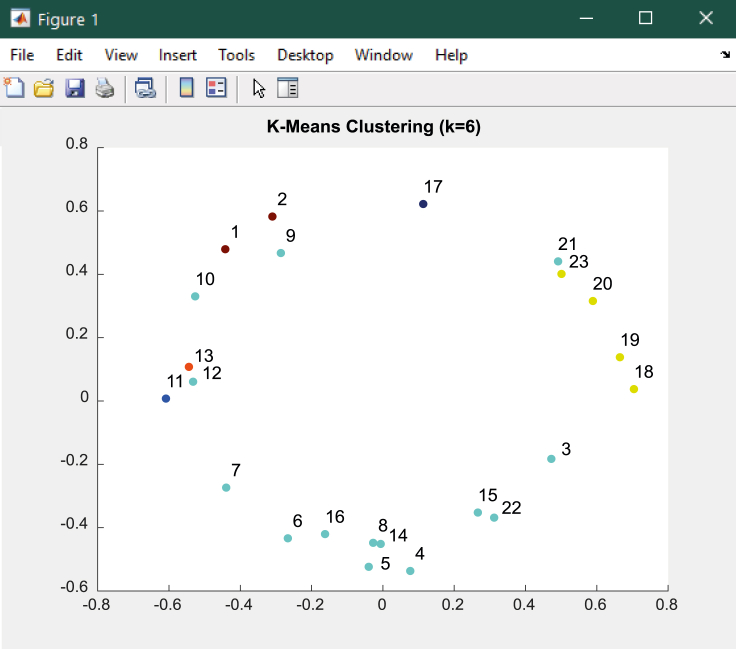
Example of *k*-means scatter plot for transcript data (*k*=6).

The purpose of clustering techniques is to partition samples into groups based on hidden patterns in data. They are particularly suitable for detecting underlying associations based on shared characteristics where there is little information available. Most clustering methods are categorized within the hierarchical and *k*-means families. On one hand, hierarchical clustering is an iterative process that progressively combines pairs of observations that are the closest in proximity until all clusters are merged within a hierarchy. On the other hand, *k*-means finds the number of clusters that minimizes the sum of squared Euclidean distances between each observation and its respective cluster mean (McLachlan et al., 2008). *K* random points in the dataset (known as cluster centroids) define the groups that the remaining data points are assigned to, which are continually relocated to the averages computed within each group until distinctive clusters are formed. When applied to transcriptomic and fluxomic data in our study, *k*-means clustering is used as a method to assess whether multi-omic datasets identify clusters of growth conditions according to their respective omic responses and which trends can be observed between growth-promoting and growth-limiting conditions. In this instance, they indicate that the single-omic datasets may benefit from being analyzed in isolation, bypassing an increase in data dimensionality that cannot be easily reduced. *k*-means clustering is run using the script *statistics_on_genes.m*, which also calls *mdscale_robust.m*, a script that applies multidimensional scaling to avoid co-location of data points during clustering. Additionally, the generation of silhouette plots (Figures 14 and 15) is used to decide the number of clusters for the final scatter plot (Figure 16).

35. We begin by loading the required variables into MATLAB:

% Load the model and transcriptomic IDs

load('SynechococcusPCC7002.mat'); %fbamodel

load('Syn7002_IDs.mat'); % list of gene IDs extracted from transcriptomic reads file

% Create a variable to store gene accession IDs from the model

genes = fbamodel.genes;

% Create a variable to store gene accession IDs from the transcriptomic datasets

genes_in_dataset = Syn7002_IDs;

% Specify the number of objectives

M = 2;

% Specify the number of variables

V = numel(genes);

36. Specify the dataset on which the clustering will be performed. The gene transcripts dataset is shown as an example, but the same steps can be repeated for all datasets (*transcripts*, *all_ATP_flux*, *ATPTF*, etc.):

% Choose dataset

all_objpairs = transcripts';

% Transpose the same dataset here

all_solutions = transcripts';

all_biomass_values = all_solutions(:,1);

37. It is important to use the transposed dataset *profiles*’ and not the original dataset *profiles*, otherwise the correlation (and all the following measures) would be computed between profiles along all the genes, instead of the correlation between genes along the profiles:

% Select the index of interest (all reactions in our case)

profiles = all_objpairs;

% Transpose profiles to compute correlation between genes

genes_vs_profiles = profiles';

38. The *zscore* function is used to standardize each of the profiles to have zero mean and unit variance, after which the *pdist* function is used to compute pairwise distances between pairs of observations in the dataset:

% Standardize profiles using zscore values and compute the pairwise distances between them

dist_correlation_vector = pdist(zscore(genes_vs_profiles), 'correlation');

% Compute the distance correlation matrix

dist_correlation_matrix = squareform(dist_correlation_vector);

***Note:****K*-means clustering requires the user to decide the number of clusters (*K*) that the data is partitioned into. Prior to clustering, different values of *K* can be tested using silhouette analysis in order to select the most suitable number of clusters for partitioning data.

39. In order to establish the optimal number of clusters, a silhouette analysis can be conducted to measure the cohesion of data points within each cluster (given by a silhouette value for each variable). The initial pre-plot in Figure 14 displays silhouette values (*y*) against the number of clusters selected (*x*), which indicates the best value to select for *K* (i.e., the number of clusters with the highest silhouette value):

prompt = 'k*−*means: Press ''y'' if the number of cluster is known, or any other key to execute silhouette analysis ';

answer = input(prompt,'s');

%

if strcmp(answer,'y')

 mean_silhouette = zeros(1,30);

 for NoClust = 2:30

 [cidx, ctrs] = kmeans(genes_vs_profiles,NoClust,'dist','correlation','rep',5,

 'disp','final');

***Note:*** Upon selecting a value for *K*, the *silhouette* function in MATLAB produces a plot (Figure 15) that displays values for each individual cluster within the range of [−1,1]. This gives a measure of proximity for each point in one cluster to points in the neighboring clusters.

% Create a silhouette plot to decide the number of clusters

 figure;

 [silh5,h] =

 silhouette(genes_vs_profiles,cidx,'corr');

 h = gca;

 h.Children.EdgeColor =

 [.8 .8 1];

 xlabel 'Silhouette

 Value';

 ylabel 'Cluster';

 end

end

40. Upon examination of the silhouette plot, the user is prompted to manually select the number of clusters for the *k*-means plot:

% Enter the number of clusters

prompt = 'k*−*means: what is the number of clusters chosen after inspection of the mean_silhouette plot?';

***Note:*** The closer the silhouette coefficients are to the value of 1, the further that point is from other clusters and the better the separation of clusters. If the point has a coefficient close to 0, this means that it is very close to the decision boundary between two neighboring clusters. After the silhouette coefficients have been calculated for data points in each cluster, a mean silhouette score can be computed to evaluate the feasibility of the entire cluster.

41. Nonmetric multi-dimensional scaling can be applied to circumvent errors caused by the co-location of data points by multiplying dissimilarities by a scalar:

% Specify the number of iterations for the scaling algorithm

options = statset('MaxIter',500);

% Perform multi*−*dimensional scaling

[Y,stress] = mdscale_robust(dist_correlation_vector,2,'criterion','sstress','start','random','Options',

 options);

***Note:****mdscale_robust* is a variation of the *mdscale* function where scaling is used to minimize the squared stress criterion with 500 iterations of the algorithm.

42. The *kmeans* function is used to perform clustering using the following command:

% Perform k-means clustering

[cidx, ctrs] = kmeans(genes_vs_profiles,num_clusters, 'dist','cityblock','rep',5,'disp','final');

***Note:*** In this instance, the ‘dist’ metric for clustering is the city block (also called “Manhattan”) distance. The formula for computing this distance can be specified in general as:

$$d_{st}=\;\sqrt[{p}]{\sum_{j=1}^{n}|x_{sj}-x_{tj}{|}^{p}}$$

where p = 1 in the case of the Manhattan distance, but the user is encouraged to choose the metric most suitable for their dataset.

43. Finally, a scatter plot can be created to display the *k*-means clusters:

% Create the final k-means plot

figure

C = cidx; %color according to k-means clustering

colormap(jet(256))

scatter(Y(:,1),Y(:,2),200,C,'.');

title(['K*−*Means Clustering (k = 'num2str(numel(unique(cidx)))')']);

labels = num2str((1:size(Y,1))','%d'); %'

text(Y(:,1),Y(:,2),labels,'horizontal','left','vertical','bottom')

### LASSO regression

**Timing: < 10 min**

The main purpose of the analysis is to identify the core subset of predictors (either genes and/or reactions) with positive or negative nonzero coefficients greater than 0.01 that are strongly related to *in vivo* growth rates by penalizing the recursive predictors (i.e., setting their coefficients to zero). The script *lasso.m* performs LASSO regression with α= 1, which returns fitted least-squares coefficients for linear models of transcript, flux or multi-omic data (*x*) and the growth rates (*y*) in 12 growth conditions. Following this, the mean predictor coefficient (MPC) can be calculated by averaging across nonzero coefficients in all vectors for each gene/reaction. In this example, only 12 out of 23 growth conditions had (i) specified growth rates, (ii) specified doubling times, or (iii) standard growth curves that could be used to calculate growth rates from the original studies (Ludwig and Bryant, 2011, 2012a,b), so only the subset of the original datasets corresponding to these growth rates has been selected for analysis. We here describe LASSO regression carried out in MATLAB for the subset of gene transcripts corresponding to these 12 growth conditions, but for the sake of clarity, the generation of multi-omic and fluxomic subsets is also demonstrated.

44. In MATLAB, create new variables which are subsets of data corresponding to the 12 conditions with available growth rates:

% Load transcripts

load('transcripts.mat')';

transcripts = transcripts';

% Specify the dimensions of the data

t_size = size(transcripts);

% Create transcript data corresponding to 12 growth conditions

transcripts_subset = ones(1,t_size(2)); % all ones for standard control

transcripts_subset(2:12,:) = transcripts([10:12,14:16,18,19,21:23],:);

% Load flux data

load('all_atp_flux.mat');

% Create flux data corresponding to 12 growth

conditions

all_atp_flux_subset = all_atp_flux([24,10:12,14:16,18,19,21:23],:);

% Load multi-omic data (concatenated transcript and flux data)

load('ATPTF.mat');

% Create multi-omic data corresponding to 12 growth conditions

ATPTF_subset = ATPTF([24,10:12,14:16,18,19,21:23],:);

% Load available growth rates corresponding to 12 growth conditions

Y2 = [0.075;0.046153846;0.05;0.035294118;0.173286795;0.266595069;0.266595069;0.038659794;

0.068807339;0.089285714;0.076530612;0.027777778];

% Create name IDs for growth conditions

Y2_names = {'Standard Control', 'N*−*limited', 'S*−*limited', 'P*−*limited', 'Nitrate',

'Ammonia', 'Urea', '22C', '30C', 'Mixotrophic', 'Low salt', 'High salt'};

45. Perform LASSO regression with each dataset acting as predictor data (*x*) and the growth rates as response (*y*):

% Perform LASSO regression

[B_transcripts,fitInfo_transcripts] = lasso(transcripts_subset,Y2);

% Average across all coefficients by finding mean of each row (predictor)

B_transcripts_mean = mean(B_transcripts,2);

% Find indices of absolute nonzero mean predictor coefficients

transcripts_abs_mean = abs(mean(B_transcripts,2));

nonzero_transcripts = find(transcripts_abs_mean > 0.01);

% Convert data into cell arrays

B_transcripts = array2table(B_transcripts);

B_transcripts_mean = array2table(B_transcripts_mean,'VariableNames',{'Mean Predictor Coefficient'});

46. Create a table that combines all data relating to nonzero predictors and their coefficients:

% Create cell array of gene IDs

transcripts_IDs = array2table([1:t_size(2)]','VariableNames',{'ID'});

% Create table of categorical data from original transcriptomic data

Dataset1 = readtable('Dataset1.xlsx');

names_transcripts = (Dataset1(:,{'LocusTag','COGCategory','CyanobaseCategory','CyanobaseSubCategory'}));

% Concatenate categorical data with B coefficients array and mean predictors

B_transcripts_table = horzcat(transcripts_IDs,names_transcripts,B_transcripts,B_transcripts_mean);

% Filter for indices with nonzero predictor coefficients > 0.01

B_transcripts_nonzero = B_transcripts_table(nonzero_transcripts,:);

% Sort coefficients in descending order

B_transcripts_zero = sortrows(B_transcripts_nonzero,{'Mean Predictor Coefficient'},{'descend'});

% Save table of coefficients as .xlsx file

writetable(B_transcripts_nonzero,'B_transcripts_nonzero.xlsx');

### Correlation analysis

**Timing: < 10 min**

**Figure 17:**
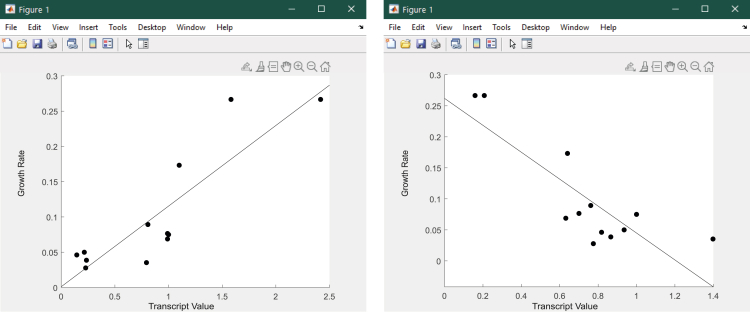
Example of PCC scatter plots for transcript data. Part of this figure is reprinted with permission from Vijayakumar et al. (2020).

This analysis indicates the strength of association between gene transcripts and/or flux values and growth rates where all flux fold changes are converted into absolute (non-negative) values prior to calculating their correlations in order to equally represent the activity of reversible reactions. Using the same data as in LASSO regression, the script *corrcoef_tf_gr.m* calculates the Pearson correlation coefficients between subsets of transcript/flux data (*x*) and growth rates (*y*) across 12 conditions. The example below demonstrates how a table of correlation coefficients calculated between the transcript data and growth rates is generated in MATLAB (*corr_transcript_table*), but the corresponding tables can also be created for flux data, i.e., *corr_ATP_table*, *corr_P1_table*, *corr_P2_table*. Example plots of the positive/negative correlation between the transcript data and growth rates are provided in Figure 17.

47. In MATLAB, create output vectors to store correlation coefficients, p-values, and lower and upper bounds of confidence intervals, changing the number of rows for transcripts (3187), fluxes (742), or both (3929):

% Create empty vectors to store outputs

corr = zeros(3187,1); % PCC

pval = zeros(3187,1); % p*−*value

lb95 = zeros(3187,1); % lower bound for 95% confidence

ub95 = zeros(3187,1); % lower bound for 95% confidence

48. Specify the size and type of dataset to be used as *x* (gene transcripts in this example):

% Specify the number of scalar observations(N)

N = size(transcripts,2);

49. A ‘for’ loop is used to iteratively calculate Pearson correlation coefficients with their respective p−values and 95% confidence intervals over the whole dataset using the *corrcoef* function:

% Calculate correlation coefficients (R) with their respective p*−*values (P) and lower and upper bounds (RL and RU) according to the 95% confidence interval:

for i = 1:N

 [R,P,RL,RU] = corrcoef(transcripts_subset(:,i),Y2); %Y2 contains growth rates

 corr(i) = R(1,2);

 pval(i) = P(1,2);

 lb95(i) = RL(1,2);

 ub95(i) = RU(1,2);

end

50. Save the data in an .xlsx table:

% Create table of correlation coefficients

corr_transcripts = array2table(corr,'VariableNames',{'PCC'});

corr_transcripts_table = horzcat(transcripts_IDs,names_transcripts,corr_transcripts);

% Sort table in descending order of PCC values

corr_transcripts_table = sortrows(corr_transcripts_table,{'PCC'},{'descend'});

% Save table of correlation coefficients

writetable(corr_transcripts_table,'corr_transcripts_table.xlsx');

51. Select data corresponding to predictors yielding the highest correlations:

% Retrieve IDs for transcripts that yield the top 10 positive PCC

top_10_positive_IDs = table2array(corr_transcripts_table([1:10],1));

% Retrieve IDs for transcripts that yield the top 10 negative PCC

corr_transcripts_table = sortrows(corr_transcripts_table,{'PCC'},{'ascend'});

top_10_negative_IDs = table2array(corr_transcripts_table([1:10],1));

% Select all data points for transcripts indexed by these top 10 PCC

transcripts_positive = transcripts_subset(:,top_10_positive_IDs);

transcripts_negative = transcripts_subset_new(:,top_10_negative_IDs);

52. Plot these predictors against the growth rates as follows:

% Create a scatter plot for the transcript with the highest positive PCC

scatter(transcripts_positive(1:12,1),Y2,'filled','black');

xlabel('Transcript Value');

ylabel('Growth Rate');

% Add a trendline

h = lsline;

h.Color = 'black';

% Create a scatter plot for the transcript with the highest negative PCC

scatter(transcripts_negative(1:12,1),Y2,'filled','black');

xlabel('Transcript Value');

ylabel('Growth Rate');

% Add a trendline

h = lsline;

h.Color = 'black';

**CRITICAL:** Examine correlation plots to check for regression artifacts (see troubleshooting problem four).

### Pathway-level correlation analysis

**Timing: < 15 min**

**Figure 18:**
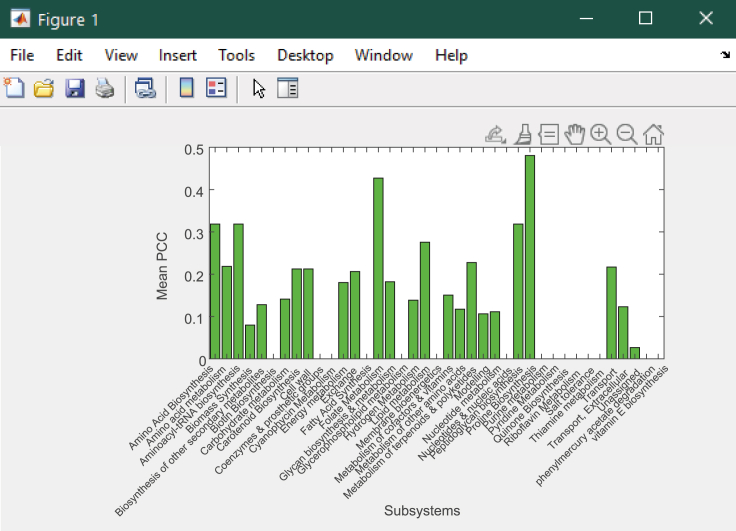
Example of bar chart for pathway-level mean absolute Pearson correlation coefficient (PCC) values calculated between Biomass - ATP maintenance fluxes (*x*) and growth rates (*y*). Part of this figure is reprinted with permission from Vijayakumar et al. (2020).

**Figure 19:**
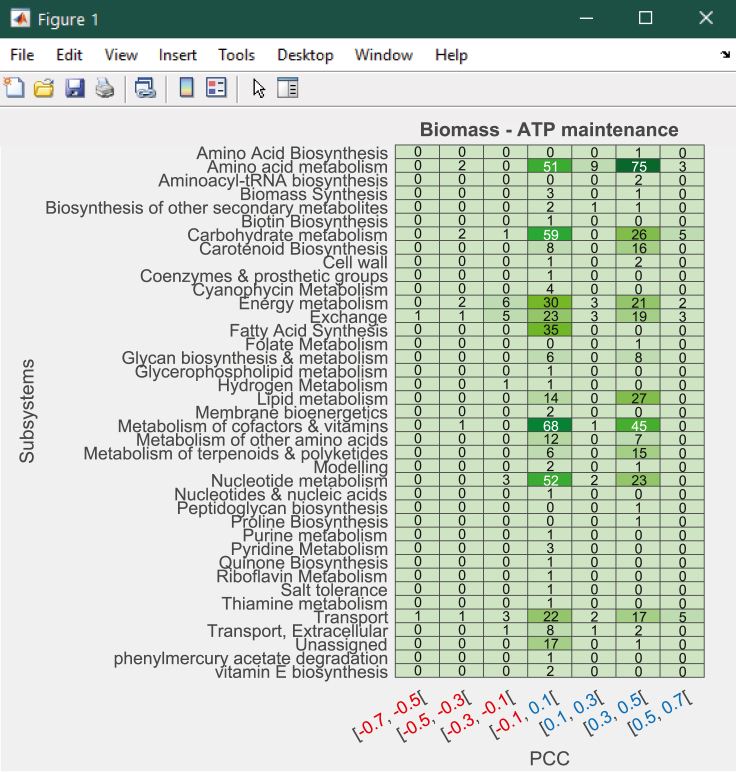
Example of PCC heatmap for pathway-level Pearson correlation coefficient (PCC) values calculated between Biomass - ATP maintenance fluxes (*x*) and growth rates (*y*) Red text in the bin labels indicates a negative correlation coefficient and blue text indicates a positive correlation coefficient. Part of this figure is reprinted with permission from Vijayakumar et al. (2020).

Similar to the pathway-level PCA, a more detailed functional classification of correlation coefficients can be yielded by performing a pathway-level correlation analysis where mean absolute PCC values are classified according to the subsystems assigned to each reaction in the GSMM (see Figure 18 for a bar plot of pathway correlations). This provides an opportunity to study these components in a more detailed manner through expanding the scope of biological insights detected and establishing connections between reactions within the same pathway. In order to account for the differing number of reactions in each pathway, the number of reactions within a binned range of PCC values can also be recorded for each subsystem listed in the model (see Figure 19 for a heatmap of pathway correlations). In this way, correlations between flux rates in each pathway and their growth rates can be assessed more fairly. In this section, we demonstrate a pathway-level analysis in MATLAB using a table of correlation coefficients calculated between Biomass - ATP maintenance flux values and growth rates (where *corr_ATP_table* has been generated using the same steps as in correlation analysis).

53. Extract correlation coefficients for the flux data in MATLAB and convert them into absolute values:

% Load PCC values from tables generated during the correlation analysis

ATP_PCC = table2array(corr_ATP_table(:,3));

ATP_PCC(isnan(ATP_PCC)) = 0;

% Convert coefficients into absolute values

ATP_PCC_abs = abs(ATP_PCC);

**CRITICAL:** Correlation coefficients are converted into absolute values prior to calculating the mean PCC for all pathways since only the magnitude of correlation (and not the direction) is considered when plotting the bar chart in Figure 18. However, the heatmap in Figure 19 indicates the signs of individual correlation coefficients as well as the number of reactions within each pathway.

54. Calculate mean PCC values for each subsystem using the same number of reactions recorded within each subsystem (*cardinality_subsystems*) and reaction indices obtained for each subsystem (*ixs_subsystems*) as in Pathway-level PCA (optional):

% Create an empty vector to store averages of PCC values for subsystems:

ATP_PCC_mean = zeros(numel(ixs_subsystems),1);

% Calculate mean PCC by subsystem

for c = 1:numel(ixs_subsystems)

 ATP_PCC_mean(c) = mean(ATP_PCC_abs(ixs_subsystems{c},1));

end

55. Plot a bar chart using the mean values:

% Set subsystem names as x*−*axis labels

X_labels = categorical(list_subsystems);

% Plot the subsystems (x) against mean pathway PCC values (y):

X = categorical(list_subsystems);

bar(X,ATP_PCC_mean);

xlabel('Subsystems');

ylabel('Mean PCC');

hold on

set(gca, 'XTickLabelRotation',45);

***Note:*** Since the mean absolute PCC values disregard the signs of individual correlation coefficients, we can also plot a heatmap recording the number of PCCs within a series of binned ranges for each subsystem. This gives a better indication of the number of reactions within each pathway as well as the direction of correlation.

56. Create variables to store PCC values for all reactions within each subsystem:

all_corr_ATP = cell(numel(ixs_subsystems),1);

% Create bins to sort PCC values

bin_1 = zeros(numel(ixs_subsystems),1);

...

bin_7 = zeros(numel(ixs_subsystems),1);

57. Use a ‘for’ loop to record the number of correlation values within a given range for each bin:

% Store correlation values for each subsystem in a cell array

for c = 1:numel(ixs_subsystems)

 all_corr_ATP{c} = ATP_PCC(ixs_subsystems{c},1);

% Within this loop, temporarily convert each row of cells into numericals

 all_corr_ATP_val = cell2mat(all_corr_ATP(c,1));

% Record the number of coefficients within each bin

 bin_1(c) = numel(find(all_corr_ATP_val >= *−*0.7 & all_corr_ATP_val < *−*0.5));

 bin_2(c) = numel(find(all_corr_ATP_val >= *−*0.5 & all_corr_ATP_val < *−*0.3));

 bin_3(c) = numel(find(all_corr_ATP_val >= *−*0.3 & all_corr_ATP_val < *−*0.1));

 bin_4(c) = numel(find(all_corr_ATP_val >= *−*0.1 & all_corr_ATP_val < 0.1));

 bin_5(c) = numel(find(all_corr_ATP_val >= 0.1 & all_corr_ATP_val < 0.3));

 bin_6(c) = numel(find(all_corr_ATP_val >= 0.3 & all_corr_ATP_val < 0.5));

 bin_7(c) = numel(find(all_corr_ATP_val >= 0.5 & all_corr_ATP_val < 0.7));

end

58. Plot the number of reactions in each bin and subsystem using a heatmap:

% Concatenate bins horizontally into an array

cdata = horzcat(bin_1,bin_2,bin_3,bin_4,bin_5,bin_6,bin_7);

% Label the bins (x)

xvalues = {'[*−*0.7, *−*0.5[','[*−*0.5, *−*0.3[','[*−*0.3, *−*0.1[','[*−*0.1, 0.1[','[0.1, 0.3[','[0.3, 0.5[','[0.5, 0.7['};

% Label the subsystems (y)

yvalues = list_subsystems(:)';

% Plot the heatmap using a custom colormap (ATPmap):

h = heatmap(xvalues,yvalues,cdata,'Title','Biomass *−* ATP maintenance','XLabel','PCC','YLabel',

'Subsystems','Colormap',ATPmap,'ColorbarVisible','off');

***Note:*** Similar heatmaps can be plotted for the Biomass - Photosystem I and Biomass - Photosystem II correlation coefficients to evaluate the correlation between metabolic flux and growth rates across various pathways.

## Expected outcomes

The main outcome of this analysis is to establish a procedure for linking specific genes and/or reactions across trans-omic layers of data belonging to the same biological system. Here we present an example of the pipeline applied to *Synechococcus* sp. PCC 7002, following the workflow laid out in Figure 3.

The process begins with tailoring the GSMM according to available transcriptomic data recorded under different conditions that influence growth and photosynthesis. After performing condition-specific FBA with norm-2 regularized bi-level optimization, comparisons can be made between the results of analyses performed upon gene transcription data, metabolic flux data and the multi-omic data resulting from their concatenation. These analyses include PCA, *k*-means clustering, LASSO regression and Pearson correlation analysis. Features identified through these analyses reflect the coordinated responses shared between different data types, as well as the variability in responses between different growth conditions. Since the flux data is informed by transcriptomic data through the integration of condition-specific growth profiles within the GSMM, the downstream effect of differential gene expression on metabolic pathways can be observed. Analyzing both transcriptomic and fluxomic data provides a more complete picture of cyanobacterial metabolism than single-omic analyses.

The protocol can be applied for numerous purposes such as model-aided discovery, hypothesis testing, identification of targets for metabolic engineering and comparison between multi-omic data across biological conditions. These processes can be optimized by examining the downstream effects of gene expression on metabolism, thereby contributing to expanding knowledge and meaningful outputs from metabolic models as well as lending biological interpretability to machine learning models. Our code and step-by-step methodology are intended to make these analyses more accessible to non-experts or serve as a guide to other investigators for combining *in silico* flux simulation with machine learning.

## Limitations

During flux balance analysis, a series of boundary constraints were defined to fine-tune the calculation of flux rates and more closely represent the metabolic capability of cells. The bounds for nutrient uptake were set based on metabolite concentrations in the growth medium, e.g. for the nitrate condition where the medium was supplemented with 12 mM of sodium nitrate, an uptake rate (lower bound) of -12 was assigned to the nitrate exchange reaction (EX_NO3_E). Currently, there exists no standard operating procedure for the definition of nutritional environments for GSMMs, as they are assessed case-by-case by researchers conducting the study (see troubleshooting problem five). A recent framework proposed a comprehensive set of guidelines in this regard, paying careful attention to the chemical composition of the growth medium as well the physiology of organism(s) concerned and various inorganic environmental factors (Marinos et al., 2020).

As photoautotrophs, cyanobacteria absorb light in excess of biomass and other maintenance requirements, which can be difficult to replicate within a GSMM. Critically, the exact photon absorbances of the *Synechococcus* sp. PCC 7002 cultures were not measured in the same conditions in which the cells were harvested for transcriptomic sequencing. Therefore, constraints for photon exchange reaction (EX_PHOTON_E) had to be approximated using values listed in literature for dry cell weight and photon absorbance for similar species and adjusted based on the availability of light for each growth condition. This process could be improved by specifying directly measured photophysiological parameters (such as light acclimation, cell density, pigment concentration, photon absorbance, oxygen evolution rate and optical density), and using these values to constrain photon uptake more accurately for each culture (Broddrick et al., 2019; Toyoshima et al., 2020).

Hence, we recommend the use of *in vivo* experimental data for various growth conditions where feasible to constrain the model and yield more precise flux rates. The prediction of internal fluxes can also be improved by using more specialized FBA techniques that consider constraints on resource allocation between biological processes, such as conditional FBA (Rügen et al., 2015), Resource Balance Analysis (RBA) (Goelzer and Fromion, 2011) or Constrained Allocation Flux Balance Analysis (CAFBA) (Mori et al., 2016).

A number of linear methods and transformations were adopted in this study to maximize the interpretability of machine learning predictions, using quadratic terms for regularization only. However, a range of techniques for dimensionality reduction or clustering methods could be implemented here, e.g. to elucidate non-linear relationships among different omics.

## Troubleshooting

### Problem 1

Raw multi-omic data originating from various sources (transcriptomic, proteomic, metabolomic) differ significantly in terms of their format and structure. Data transformation, normalization or scaling techniques must be applied as forms of pre-processing prior to integration in order to make these data comparable. Particularly, the batch effect must be taken into account both before and after conducting experiments since this gives rise to unwanted variation in datasets caused by differences in technical factors across batches (Step 10 of before you begin).

### Potential solution

Methods such as ComBat allow users to adjust for batch effects among samples by utilizing parametric or non-parametric empirical Bayes frameworks (Johnson et al., 2007; Zhang et al., 2020b). Other techniques such as SVASeq or RUVSeq also help to eliminate noise from sequencing experiments and adjust for technical interference (Leek, 2014; Risso et al., 2014). These would be followed by the pre-processing steps. If available, integrating proteomic or metabolomic data into a GSMM can provide a more accurate representation of the cellular phenotype since they include effects downstream of genes and gene transcripts.

### Problem 2

There are numerous methods available for integrating multi-omic data within GSMMs, and it can be challenging to choose a single method for data integration (Step 7 of step-by-step method details).

### Potential solution

There are many types of approaches to consider for multi-omic data integration, several of which are discussed elsewhere in greater detail (Machado and Herrgård, 2014; Cho et al., 2019).

In summary, the generation of context-specific metabolic models is divided into two main classes: (i) switch-based approaches (such as GIMME), which remove inactive or lowly expressed genes by setting the corresponding reaction boundaries to zero, and (ii) valve-based approaches (such as E-flux), which increase or decrease the activity of highly (or lowly, respectively) expressed genes by adjusting the upper and lower bounds for their corresponding reactions, proportional to their normalized gene expression values (Vijayakumar et al., 2018).

The main advantage of GIMME-like methods is that they can re-enable flux associated with false negative values in inactive reactions and record consistencies between gene expression data and flux predictions. On the other hand, non-discretized relative gene expression values are more indicative of protein concentrations since levels of transcription are more comparable across genes. The approach used in this case study is closer to a valve-based approach based on METRADE (Angione and Lió, 2015), where the expression level of each gene set (represented by the vector ϴ) is mapped to a coefficient for the lower and upper bounds of the corresponding reaction in the GSMM. When using our method, it was important to conduct a sensitivity analysis to select the optimal value for the γ parameter, which magnified the level of gene upregulation or downregulation and therefore the metabolic sensitivity for yielding experimentally feasible flux values for different growth conditions.

In addition to switch- and valve-based integration methods, there are alternative methods that consider the cellular goal specific to each GSMM or remove unnecessary/blocked reactions from the network. Metabolic task derived (MTD) algorithms consider the main objective function(s) that represent the metabolic tasks as the main priority for the cell or community or utilize omics-guided objective functions, as in omFBA (Guo and Feng, 2016). Network-pruning methods (such as MBA) retain only a core set of reactions in the network by iteratively pruning reactions from the model to derive a sub-network that is consistent with the tissue-specific gene expression, among other data. However, these methods are only used to extract a context-specific model and do not provide a corresponding flux distribution. Therefore, the method chosen for data integration depends on the nature of the data, the approach taken for constraining flux bounds and the optimization problem to be solved. During model extraction, the type of thresholding applied (within samples or genes) and the threshold values for gene expression used can also affect the output models (Walakira et al., 2021). Very few methods automate model extraction and flux prediction without *a priori* knowledge of context-specific functions or binarization of reactions during data integration. However, RegrEx is one such algorithm that uses regularized least-squares optimization for automated model extraction and unbiased flux calculation (Robaina Estévez and Nikoloski, 2015).

### Problem 3

The cut-off value for setting fluxes equal to zero (10^−4^) may not be applicable for every model, seeing as fluxes toward biomass building blocks and other important metabolic components are at risk of being eliminated (Step 13 of step-by-step method details).

### Potential solution

We advise users of the protocol to conduct a robustness analysis to assess different thresholds for flux and fold change values. Starting from the solver tolerance parameter (10^−6^ in our case), we recommend increasing the order of magnitude for setting flux rates to zero until a trade-off can be reached between eliminating noise within the data whilst still retaining the ability to identify and quantify functionally significant contributions of metabolic processes. Values that are below the chosen threshold can then be set to zero based on this adjustment without any statistically significant changes in results.

### Problem 4

The correlation analysis may give rise to regression artifacts that do not reflect a true linear correlation between gene transcript/flux data and growth rates, leading to incorrect causal inferences (Step 52 of step-by-step method details).

### Potential solution

We advise users to manually inspect each correlation plot to assess the validity of correlation between variables. Alternatively, there are preprocessing techniques that can be applied to data such as global scaling normalization or dropout imputation. In some instances where artifacts have been introduced as a result of data oversmoothing or overfitting, reintroducing random noise into datasets has been shown to increase robustness (Zhang et al., 2021).

### Problem 5

There is no standard operating procedure for determining uptake rates (Step 4 of step-by-step method details).

### Potential solution

In the absence of *in vivo* uptake rates obtained from time-course metabolomic experiments, we advise users to approximate uptake rates, starting from the concentration of the organic carbon source in the growth medium (e.g., glucose or glycerol) and convert these values into flux units mmol/gDW h^-1^ (Schinn et al., 2021). Methods such as Metabotools already use extracellular concentrations to calculate and adjust constraints by defining growth media in terms of concentrations of metabolites measured in mM (Aurich et al., 2016).

Although inorganic substrates are not usually constrained, the inorganic carbon uptake rate is accepted in the absence of a carbon substrate for photoautotrophic organisms such as cyanobacteria (Qian et al., 2017). Furthermore, as the availability of nutrients has a major impact on the calculation of metabolic fluxes, we incorporate the extracellular concentrations of metabolites and co-factors present within various growth media for different conditions to constrain the lower and upper bounds of the associated exchange reactions in the model. This application of condition-specific constraints on the exchange reactions ensures that exchange rates emulate uptake and secretion of metabolites in accordance with the experimental data and the computational model therefore more closely resembles the experimental conditions in which the cells are cultured.

## Resource availability

### Lead contact

Further information and requests for resources should be directed to and will be fulfilled by the lead contact, Claudio Angione (c.angione@tees.ac.uk).

### Materials availability

The study did not generate new unique reagents or other materials.

## Data Availability

This protocol fully specifies all datasets generated or analyzed during the study. The complete source code relating to all procedures listed within the protocol is freely available on GitHub at: https://github.com/Angione-Lab/Synechococcus7002-metabolic-modelling.
